# Human Sentinel Surveillance of Influenza and Other Respiratory Viral Pathogens in Border Areas of Western Cambodia

**DOI:** 10.1371/journal.pone.0152529

**Published:** 2016-03-30

**Authors:** Ans Timmermans, Melanie C. Melendrez, Youry Se, Ilin Chuang, Nou Samon, Nichapat Uthaimongkol, Chonticha Klungthong, Wudtichai Manasatienkij, Butsaya Thaisomboonsuk, Stuart D. Tyner, Sareth Rith, Viseth Srey Horm, Richard G. Jarman, Delia Bethell, Nitima Chanarat, Julie Pavlin, Tippa Wongstitwilairoong, Piyaporn Saingam, But Sam El, Mark M. Fukuda, Sok Touch, Ly Sovann, Stefan Fernandez, Philippe Buchy, Lon Chanthap, David Saunders

**Affiliations:** 1 Department of Immunology, US Armed Forces Research Institute of Medical Sciences, Bangkok, Thailand; 2 Viral Diseases Branch, Walter Reed Army Institute of Research, Silver Spring, Maryland, United States of America; 3 Department of Immunology, Armed Forces Research Institute of Medical Sciences, Battambang, Cambodia; 4 Department of Virology, Armed Forces Research Institute of Medical Sciences, Bangkok, Thailand; 5 Virology Department, Institut Pasteur du Cambodge, Phnom Penh, Cambodia; 6 Deputy Director, Armed Forces Health Surveillance Center, Silver Spring, Maryland, United States of America; 7 Communicable Disease Control Department, Ministry of Health, Phnom Penh, Cambodia; 8 Department of Immunology, Armed Forces Research Institute of Medical Sciences, Phnom Penh, Cambodia; University Hospital San Giovanni Battista di Torino, ITALY

## Abstract

Little is known about circulation of influenza and other respiratory viruses in remote populations along the Thai-Cambodia border in western Cambodia. We screened 586 outpatients (median age 5, range 1–77) presenting with influenza-like-illness (ILI) at 4 sentinel sites in western Cambodia between May 2010 and December 2012. Real-time reverse transcriptase (rRT) PCR for influenza was performed on combined nasal and throat specimens followed by viral culture, antigenic analysis, antiviral susceptibility testing and full genome sequencing for phylogenetic analysis. ILI-specimens negative for influenza were cultured, followed by rRT-PCR for enterovirus and rhinovirus (EV/RV) and EV71. Influenza was found in 168 cases (29%) and occurred almost exclusively in the rainy season from June to November. Isolated influenza strains had close antigenic and phylogenetic relationships, matching vaccine and circulating strains found elsewhere in Cambodia. Influenza vaccination coverage was low (<20%). Western Cambodian H1N1(2009) isolate genomes were more closely related to 10 earlier Cambodia isolates (94.4% genome conservation) than to 13 Thai isolates (75.9% genome conservation), despite sharing the majority of the amino acid changes with the Thai references. Most genes showed signatures of purifying selection. Viral culture detected only adenovirus (5.7%) and parainfluenza virus (3.8%), while non-polio enteroviruses (10.3%) were detected among 164 culture-negative samples including coxsackievirus A4, A6, A8, A9, A12, B3, B4 and echovirus E6 and E9 using nested RT-PCR methods. A single specimen of EV71 was found. Despite proximity to Thailand, influenza epidemiology of these western Cambodian isolates followed patterns observed elsewhere in Cambodia, continuing to support current vaccine and treatment recommendations from the Cambodian National Influenza Center. Amino acid mutations at non-epitope sites, particularly hemagglutinin genes, require further investigation in light of an increasingly important role of permissive mutations in influenza virus evolution. Further research about the burden of adenovirus and non-polio enteroviruses as etiologic agents in acute respiratory infections in Cambodia is also needed.

## Introduction

Acute respiratory infection (ARI) is the leading cause of morbidity and mortality in Cambodia [1]. Previous studies have attributed the etiology of acute viral respiratory infections in Cambodia to rhinovirus, respiratory syncytial virus (RSV), parainfluenza virus (PIV), influenza virus A and B, human metapneumovirus (HMPV), bocavirus, adenovirus, enterovirus, and coronavirus [2]. Influenza has been described to be one of the most important causes for hospitalization of children with an ARI [3]. It was suggested that some Southeast Asian countries, where influenza A infection is present year round, play an important role in the global spread of influenza that could trigger annual epidemics in temperate regions [4,5].

Until the establishment of the National Influenza Center (NIC) at Institut Pasteur du Cambodge (IPC) and a national influenza-like-illness (ILI)-sentinel surveillance system in 2006, little was known about influenza circulation in Cambodia. Since then, the sentinel surveillance system has, together with event-based surveillance, demonstrated evidence of seasonal, pandemic and avian influenza. Previously published data revealed that Thailand and Cambodia, which are positioned geographically in the Northern hemisphere, demonstrate differing influenza epidemiology with regard to seasonality, with Thailand exhibiting a northern hemisphere transmission pattern while transmission in Cambodia typically occurs from June to December, similar to the southern hemisphere [6]. The rainy season in Western Cambodia is June to November and the dry season is December to May. The more recent data suggests that influenza seasonality is more similar in Thailand and Cambodia than previously thought. [5].

Depending on the type of genes and the specific residues, phenotypic effects of random or neutral mutation of the virus can be positive, purifying or neutral [7]. Influenza virus efficiently escapes from host antibodies through an accumulation of mutations/single amino acid changes (antigenic drift) at the antigenic sites (epitopes) in surface glycoproteins of the hemagglutinin (HA) gene, and to a lesser extent, neuraminidase (NA) genes [8]. The antigenic sites are five somewhat overlapping regions (designated A to E for the H3 strains [9–11] and Ca1, Ca2, Cb, Sa, and Sb for the H1 strains [12]). Antigenic mapping by Smith et al. [13] showed that 11 antigenic clusters of viruses emerged during the 35-year period following the introduction of the A/H3N2 virus in humans in 1968 and that major jumps (or "cluster transitions") occur between antigenically distinct clusters of viral sequences roughly every 3 years (antigenic shift) [13].

Until 2009, the Cambodian national sentinel surveillance system only covered 8 out of 24 provinces based on a network of urban sentinel-sites at referral hospitals. No data existed about circulating influenza strains and other respiratory pathogens in Western-Cambodia along the Thai-Cambodia border, which is a poor and rural area with large volume of cross-border traffic. Between 2010 and 2012, the Armed Forces Research Institute of Medical Sciences (AFRIMS) established four additional influenza sentinel surveillance sites in four border provinces in western Cambodia, in collaboration with the Cambodian Communicable Disease Control (CCDC) Department and the IPC.

This study describes the circulation of influenza and non-influenza respiratory viruses and the genetic diversity of influenza viruses in western Cambodia along the Thai-Cambodia border which has provided information for treatment, prevention, and control strategies for these populations.

## Materials and Methods

### Ethics

The ILI and event-based surveillance systems are public health activities organized by the Ministry of Health in Cambodia and as such have a standing authorization from the National Ethics Committee. Samples were all anonymized for the purpose of this study. The study was approved by Scientific Review Committee at AFRIMS in Thailand, the Institutional Review Boards at the Walter Reed Army Institute of Research (WRAIR) in the United States and the National Ethics Committee for Health Research (NEHCR) in Cambodia. Written informed consent was obtained from volunteers or parents or legal guardians of children enrolled in the study.

### Study design

**Fig 1:**
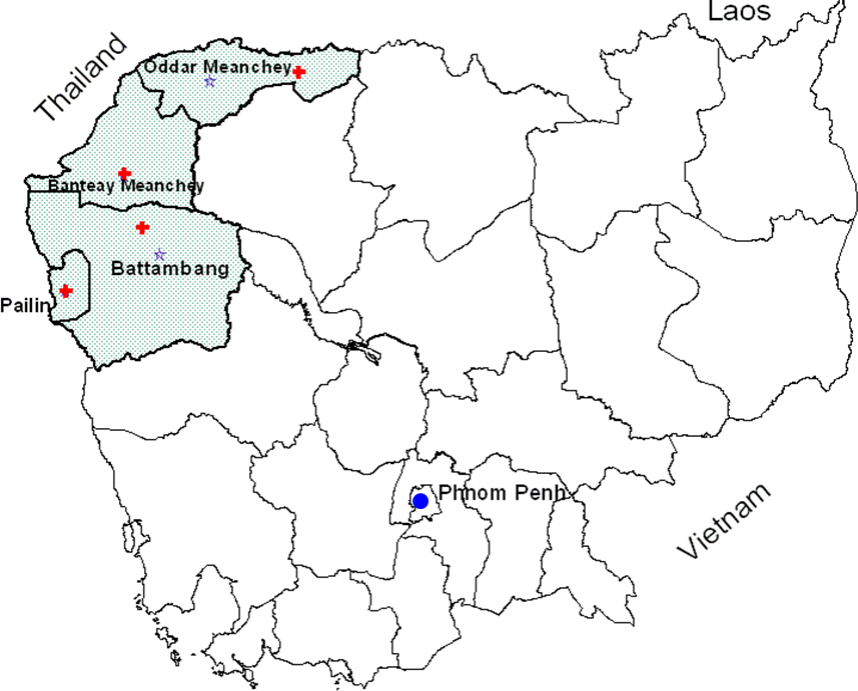
Locations of influenza-like-illness (ILI)-sentinel sites in Cambodia. Study area is shaded; sentinel sites are indicated by a plus symbol in red and field laboratories are indicated by purple stars.

Between May 2010 and December 2012, we collected specimens and surveillance data for influenza and other viral respiratory pathogens from a subset of outpatients presenting with influenza-like-illness (ILI) at four sentinel sites-located in five health centers and hospitals in Battambang, Oddar Meanchey, Pailin and Banteay Meanchey provinces in Cambodia (Fig 1). Fig 1 was created using ArcView GIS version 3.1 (http://arcview.software.informer.com [14,15])

Sentinel health centers and hospitals were selected based on sufficient overall patient volume and patients with upper respiratory infections (URI), geographical representativeness and ease of specimen transport to the study laboratory. Sites were established sequentially: Battambang in 2010 (2 sites), Oddar Meanchey and Pailin in 2011, and Banteay Meanchey in 2012. Due to the cross-sectional design of our study in a region for which no previous baseline respiratory surveillance existed, no minimal sample size was calculated. Nevertheless, and in accordance with national and World Health Organization influenza surveillance guidelines [16], we aimed to collect a cross-section of data distributed over time and space. For every site -and pending on local epidemiology and health facility workload, 5 to 10 ILI specimens were collected each week (Monday to Friday) for 52 weeks per year and largely from the first two subjects meeting the predefined inclusion criteria.

Demographic, clinical data and respiratory specimens were collected from patients who met the case definition for ILI defined as persons of 1 year of age or older, arriving at the health center or hospital within 5 days of fever onset with a fever (axillary ≥38.1°C or oral ≥ 38.6°C) and cough or sore throat in the absence of other diagnoses. Patients younger than 1 year, who could not provide consent or those with nasal lesions or epistaxis were excluded. Demographic data included gender, occupation, presenting signs and symptoms, self-reported influenza vaccination history, medication use and travel within the last 7-days. Parents reported for children, which in our study are defined as all study volunteers younger than 18 years old.

Descriptive statistics of demographic and clinical data including frequencies and cross-tabulations were entered in a study database by double data-entry using Microsoft Excel 2013 and analyzed using IBM SPSS Statistics 21.0 (SPSS, IBM Inc.)

### Specimen collection

Up to three respiratory swabs per volunteer were collected by trained staff with standard procedures. One nasal swab was tested on-site with QuickVue Influenza A+B (Quidel, San Diego, Calif.) using monoclonal antibodies specific for influenza A and B virus. A combined nasal and throat specimen was placed in a universal transport medium (UTM, Copan Diagnostics, Corona, CA, USA), stored between 2–8°C or in liquid nitrogen and shipped to the AFRIMS-CNM (Cambodia National Malaria Center) laboratory at the Battambang Referral Hospital for testing for influenza virus.

### Laboratory methods

All ILI-specimens were typed and subtyped for influenza A and B at the AFRIMS-CNM laboratory in Battambang. Influenza-positive specimens were forwarded to the Virology Unit at Institut Pasteur du Cambodge (IPC) in Phnom Penh and the WHO Collaborating Center (WHOCC) for Reference and Research on Influenza in Melbourne, Australia. Nucleotide sequence and phylogenetic analysis of influenza viruses was conducted at the Virology Branch at the Walter Reed Army Institute of Research (WRAIR), Silver Spring, Maryland, U.S. Influenza-negative specimens were sent to the Department of Virology at AFRIMS in Bangkok, Thailand for viral culture and further characterization of other respiratory viruses.

### Testing for influenza virus

RNA was extracted from an aliquot of 140 μl of each combined nasal and throat specimen using Qiagen Viral RNA mini kits (Qiagen, Hilden, Germany). The influenza genome was amplified and detected using a standardized the real-time reverse transcription (rRT)-PCR assay on a SmartCycler® II platform (Cepheid, Sunnyvale, CA), to test for influenza A and/or B types. Samples positive for influenza A and/or B were further tested to confirm influenza A and/or B positivity and to determine influenza A subtypes.

The hemagglutinin-gene (HA) specific primers and probes were used for subtyping of human influenza A subtypes H1, H3, H5 and pandemic H1 (pH1, previously known as swH1). Nucleoprotein-gene (NP) specific primers and probe for universal swine influenza was also used to confirm influenza A subtype pH1 detection in some samples. The 25 μl reaction mixture consisted of 5 μl of RNA eluate and 20 μl of the master mixture which included 0.5 μl enzyme mixture (SuperScript III RT/Platinum® Taq DNA Polymerase-Life Technologies), 0.5 μl RNaseOUT™ Recombinant Ribonuclease Inhibitor (InVitrogen™, Carlsbad, CA), 12.5μl 2X Reaction Mix, 0.5μl of both primers and 0.5μl of probe, and 5μl RNase-free water (see S1 Table for primer sequences). Thermocycling reaction conditions for influenza typing were as follows: initial reverse transcription at 50°C for 30 min, followed by denaturation and PCR activation by Taq inhibitor activator at 95°C for 2 min and 45 cycles of PCR amplification (<95°C for 15 sec then 64°C for 30 sec). For influenza A subtyping: reverse transcription at 50°C for 30 min, PCR activation by Taq inhibitor activator at 95°C for 2 min and 45 cycles of PCR amplification (<95°C for 15 sec then 62°C for 30 sec). After influenza typing by rRT-PCR, two 500 μl aliquots of every influenza-positive specimen was transferred into cryogenic vials for transport in liquid nitrogen to IPC in Phnom Penh.

### Definitive diagnostics for influenza

Specimens testing positive for influenza by rRT-PCR were inoculated onto Madin-Darby canine kidney (MDCK) cells for isolation of influenza strains at the Virology Unit of IPC. The influenza strains were analyzed by a hemagglutination inhibition test using reference antigens and anti-sera provided by the WHOCC for Reference and Research on Influenza in Melbourne, Australia. The NA-Star® kit (Life Technologies, Carlsbad, CA, USA), a chemiluminescent neuraminidase (NA) inhibition assay which utilizes a 1,2-dioxetane derivative of sialic acid as substrates, was used for NA inhibitor susceptibility testing on a subset of isolates, randomly selected across specimen collection sites and dates. The concentration of drug required to inhibit 50% of the NA activity (IC_50_) was calculated using the non-linear curve-fitting function in the Graphpad Prism 4 package (GraphPad Software, Inc., La Jolla, CA, USA). The average IC_50_ (nM) (± standard deviation) of two independent determinations was calculated for each virus. Outliers of more than 2 standard deviations from the overall mean were retested twice. A subset of isolates were forwarded to WHOCC in Melbourne for confirmation of strain analysis by HA inhibition test and NA inhibitor susceptibility using an NA enzyme inhibition assay with a fluorescent substrate MUNANA [2’-(4-Methylumbelliferyl)-α-D-N-acetylneuraminic acid sodium salt hydrate]. The typical range of IC_50_ was calculated as the mean IC_50_ ± 3 standard deviations using a panel of well-characterized reference strains kindly provided by the WHO Collaborative Center for Reference and Research on Influenza, Melbourne, Australia. Isolates with IC_50_ values within or close to the typical IC_50_ range were considered to be sensitive isolates. IC_50_ values outside of the typical range and between 50 and 200 nM were isolates with mildly reduced sensitivity and IC_50_ values well outside the typical range and greater than 200 nM were considered as isolates with highly reduced sensitivity.

### Nucleotide sequence analysis and phylogenetics of influenza viruses

Viral RNA segments, extracted from MDCK supernatant were sequenced at IPC in Phnom Penh from 15 samples collected in 2011 and from 10 samples collected in 2012 for both pandemic influenza (pH1N1) and influenza H3N2 (H3N2), representing our four study sites with varying success (S2 Table). Cleaned nucleotide sequence data was sent in FASTA-format to the Bioinformatics Section at the Virology Branch of the WRAIR in Silver Spring, Maryland, USA.

Sequences for pH1N1 and H3N2 sequence datasets respectively were combined with references available from the Influenza Viral Resource at Genbank [17] and the GISAID EpiFlu database [18] representing global diversity of pH1N1 from 2009–2013. Sequences were aligned using MUSCLE version 3.8.31 [19] implemented in Seaview version 4.4.2 [20] and manually inspected for accuracy. Best models were determined from alignments using jmodeltest2 version 2.1.4 [21]. Maximum likelihood (ML) phylogenetic trees were generated using PhyML version 3.0 as implemented in Seaview version 4.4.2 [22]. See Table 1 for a detailed description of datasets and models used in analysis. ML phylogenies were annotated using MEGA version 5 and FigTree version 1.4.0 [23]. All influenza sequences used in analysis have been deposited into Genbank under accessions KU299790—KU299957.

**Table 1:** Dataset descriptions and models used in maximum likelihood phylogenetic analysis.

Influenza	Segment	Full/	No. of	Specimen collection by site and year								No. of References	Model^c^	-lnL
Subtype		Partial	Samples	BTB		OM		PL		BMC				
				2011	2012	2011	2012	2011	2012	2011	2012			
pH1N1	HA	Partial^a^	14	3	1	7	0	1	1	0	1	201	GTR+G	7486.9
pH1N1	NP	Full	15	3	2	7	0	1	1	0	1	125	GTR+G	3852.9
pH1N1	NA	Partial^b^	14	3	1	7	0	1	1	0	1	125	GTR+G	4081.0
pH1N1	MP	Full	15	3	2	7	0	1	1	0	1	124	GTR+I	2212.4
pH1N1	NS	Full	15	3	2	7	0	1	1	0	1	125	GTR+I	2292.2
				**5**^d^		**7** ^d^		**2** ^d^		**1** ^d^				
H3N2	HA	Full	10	2	1	1	5	1	0	0	0	169	GTR+G+I	8028.8
H3N2	NA	Full	10	2	1	1	5	1	0	0	0	103	GTR+G	4569.2
H3N2	MP	Full	10	2	1	1	5	1	0	0	0	122	GTR+G	3156.4
H3N2	NS	Full	10	2	1	1	5	1	0	0	0	110	GTR+I	3325.5
				**3** ^d^		**6** ^d^		**1** ^d^		**0** ^d^				

BTB: Battambang, OM: Oddar Meanchey, PL: Pailin, BMC: Banteay Meanchey.

^a^ Covering nucleotides 22–1683 of the HA gene.

^b^ Covering nucleotides 97–1396 of the NA gene.

^c^ As determined by jmodeltest2 version 2.1.4 (http://code.google.com/p/jmodeltest2/).

^d^ Total number of samples from site.

### Sequence Statistics, Amino Acid Analysis and Selection Tests

Alignments were analyzed for diversity, percent nucleotide similarity, antigenic variation and selection using MEGA version 5 [24] and the HyPhy package [25] implemented through the DataMonkey webserver [26–28]. Annotation was conducted using Genbank’s annotation tool in the Influenza Viral Resource [17] and a literature review.

### Testing for non-influenza respiratory viruses at AFRIMS, Bangkok

**Fig 2:**
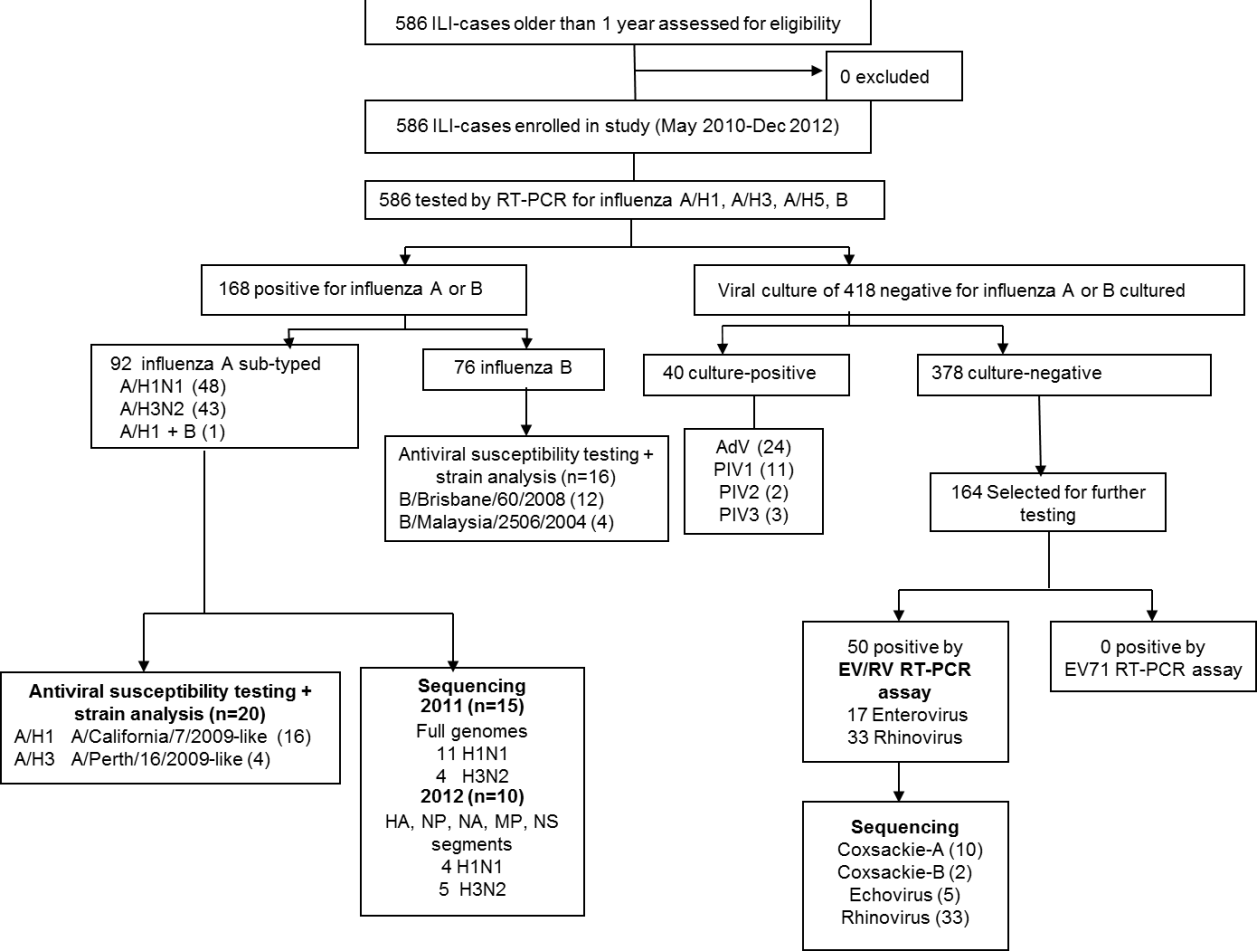
Laboratory testing flowchart for influenza-like-illness (ILI).

All specimens tested negative by influenza PCR were cultured for isolation of respiratory virus and detection by cytopathic effect (CPE) and/or with either immunofluorescence assay (IFA), or direct fluorescence assay (DFA). A subset of 164 culture-negative specimens (collected between May 2010 and April 2012), where we found a higher proportion (5.6%) of non-polio enteroviruses in children less than 5 years old as compared with previous studies (1%) in Cambodia [2], were tested for enterovirus and rhinovirus by two separate nested RT-PCR methods adapted from Coiras et al., 2004 and Singh et al., 2002 [29,30], one for simultaneous detection of pan-enteroviruses and rhinoviruses, and the other specific for enterovirus 71 (EV71). PCR-products positive for enterovirus or rhinovirus were sequenced for nucleotide analysis (Fig 2; S3 Table).

### Cell culture of influenza-negative specimens

Patient specimens in UTM were inoculated into three cell lines for virus isolation: Madin-Darby canine kidney (MDCK), Human lung carcinoma (NCI-H292) and Rhesus monkey kidney (LLCMK2) cells. Upon the appearance of CPE, after 7–10 days of culture, or after three passages, the cells were spotted onto microscope slides. Cell suspensions were dried and fixed in chilled acetone for 15 minutes. IFA was performed to identify virus isolates. The Respiratory Virus Screening and Identification Kit (Light Diagnostics, Respiratory Panel 1, Viral Screening and Identification IFA, Millipore Corporation, MA, USA) was utilized for the identification of adenoviruses, influenza A, influenza B, PIV (types 1, 2, and 3), and RSV.

### Enterovirus 71 (EV71) nested RT-PCR

Enterovirus EV71 detection included single-step first-round RT-PCR and semi-nested PCR (primers are in S3 Table). The EV71 type specific primers were modified from the previous study by Singh et al. 2002 [30]. The modifications were made by following the alignments of VP1 sequences of different EV71 strains collected during 2002–2011 from Thailand, Taiwan, Philippines, Vietnam, and China available in Genbank including the sequences with the accession No. JN191177-9, FJ969151, FJ969163, JQ621835, JQ621841, AM490141-63, JQ315092, and JX203305. Single-step first-round RT-PCR was performed in following mix: 5 μl RNA suspension, 5 μl of 10x PCR buffer II supplied with AmliTaq^TM^ DNA polymerase (Applied Biosystems), 0.2 mM of each dNTP, 1.5 mM MgCl_2_, 0.25 pmol of each forward and reverse primer (EV71-VP1f and EV71-VP1r), 5 mM dithiothreitol (DTT), 1 U reverse transcriptase from avian myeloblastosis virus (AMV RT, Promega, Madison, WI), 1.25 U of AmpliTaq^TM^ DNA polymerase (Applied Biosystems) in a final volume of 50 μl. The reverse transcription (RT) step was performed at 42°C for 60 min, followed by 35 cycles of thermocycling 94°C for 30 seconds, 50°C for 30 seconds, and 72°C for 30 seconds.

### Pan-enteroviruses and rhinoviruses nested RT-PCR

Detection of pan-enteroviruses and rhinoviruses (pan-EV/RV nested RT-PCR) is similar to the procedure for EV71 including 2 rounds of PCR as reported before [29]. The primers for simultaneous detection of enteroviruses and rhinoviruses were designed in the polyprotein gene, between 5′ non-coding region (5′NCR) and VP4/VP2 regions that was previously described by Coiras et al. 2004 [29]. EV/RV-2n was modified from primer 2-EV/RV [29] for using in nested PCR reaction. Single-step first-round RT-PCR reaction was the same as described above except that 0.25 pmol of each forward and reverse primer (1-EV/RV and 2-EV/RV) was used (S3 Table). The RT step was performed at 42°C for 60 min, followed by 35 cycles of thermocycling 94°C for 30 seconds, 50°C for 1 min, and 72°C for 1 min. The reaction was further incubated at 72°C for 10 min. Five micro-liter of 1:20 diluted first round PCR products were added to the nested PCR reaction with the same reagents as in the first-round but without DTT and AMV-RT and the primers were 0.25 pmol of each forward and reverse primer, 3-EV/RV and EV/RV-2n. The reaction was incubated at 95°C for 5 min followed by 35 cycles of thermocycling 94°C for 30 seconds, 52°C for 1 min, and 72°C for 1 min. The PCR products (9 μl each) were subjected to electrophoresis in agarose gels, with 100-bp DNA ladder serving as a molecular marker. A negative control of RNA-free water and a positive control of cDNA template from the EV71 reference strain (ATCC^®^ No. VR-1432^TM^) were included in each experiment.

### Nucleotide sequence analysis of pan-enteroviruses and rhinoviruses

The PCR products from the positive samples were sequenced on both strands with the PCR primers (S3 Table). The specific bands from the first or second rounds PCR were purified using QIAquick Gel Extraction Kit (Qiagen, Germany) before sending for direct sequencing. Sequencing service was performed by AIT biotech (Singapore). The sequences from both strands were combined for analysis and edited with Sequencher (Gene Code Corp., USA). Homology searches were through nucleotide BLAST program [31] along with the percentage of sequence identity of the two given sequences.

## Results

586 ILI-patients (median age 5 year, range 1–77) were enrolled from five sites from May 2010 to December 2012; among these, 168 (29%) tested positive for influenza by rRT-PCR (Table 2). Most frequent symptoms reported were fever (100%, inclusion criterion), cough (100%,), runny nose (90%), congestion (77%), sore throat (75%,) and headache (76%). Although gender distribution was similar in ILI-patients, more females were positive for enterovirus (71%) or rhinovirus (64%). Patients with influenza were slightly older (median age 7 years old) and presented more often with a sore throat (78%). Compared with other ILI-cases, patients testing positive for enterovirus reported body pain (20% vs. 44%) and headache (50% vs. 77%) less frequently, although chills (50% vs. 32%), vomiting (35% vs. 16%) and abdominal pain (29% vs. 19%) were more common. Dyspnea was uncommon, yet most often seen in patients positive for adenovirus (14%). Diarrhea was observed in ILI-patients (6%-12%) except those infected with enterovirus (0/17).

**Table 2:** General characteristics of the study population.

		ILI cases (n = 586)		Influenza (n = 168)		Enterovirus (n = 17)		Rhinovirus (n = 33)		Adenovirus (n = 24)	
		No.	%	No.	%	No.	%	No.	%	No.	%
**Sex**	Male	281	48	93	55	5	30	12	36	14	59
	Female	305	52	75	45	12	71	21	64	10	42
**Age (yrs)**											
	Mean, ± SD	8.8±10.6		11.2±11.2		2.7±1.5		3.8±3.0		4.3±9.5	
	Median, [range]	5 [1–77]		7 [1–72]		2 [1–6]		3 [1–15]		2 [1–48]	
	0–4 yrs*	248	42	42	25	14	82	20	61	19	79
	5–17 yrs	260	44	94	56	3	18	13	39	4	17
	18–49 yrs	73	13	30	18	0	0	0	0	1	4
	≥ 50 yrs	5	1	2	1	0	0	0	0	0	0
**Site**	Thmor Koul **(BTB)	107	18	37	22	2	12	5	15	2	8
	Tapoung **(BTB)	184	31	47	28	12	71	14	42	5	21
	Anlong Veng (OM)	231	39	62	37	3	18	13	39	15	63
	Pailin (PL)	46	8	14	8	0	0	1	3.0	2	8
	Preah Punlear (BM)	18	3	8	5	0	0	0	0		
**Occupation**											
	Child/Student	508	87	135	80	17	100	33	100	23	96
	Farmer	36	6	13	8	0	0	0	0	1	4
	Housewife	15	3	8	5	0	0	0	0	0	0
	Unemployed	5	1	2	1	0	0	0	0	0	0
	Laborer	11	2	6	4	0	0	0	0	0	0
	Government	10	2	3	2	0	0	0	0	0	0
	Other	1	0.2	1	1	0	0	0	0	0	0
**Travel last 7 days**		36	6	12	7	2	12	2	6	2	8
**Temperature (axillary-in °C)**											
	Mean, ± SD	38.7±0.5		38.8±0.5		38.7±0.5		38.6±0.4		38.7±0.5	
	Median, [range]	38.6 [38–41]		38.6 [38–41]		38.5 [38–40]		38.5 [38–40]		38.5 [38–40]	
**Signs and symptoms**											
	Sore throat	343/460	75	120/153	78	3/6	50	12/21	57	4/12	33
	Cough	583/586	99.5	168/168	100	17/17	100	33/33	100	24/24	100
	Runny nose	529/586	90	153/168	91	17/17	100	29/33	88	24/24	100
	Congestion	422/552	77	119/162	73	7/14	50	26/33	79	20/21	95
	Difficulty breathing	44/563	8	12/163	7	1/16	6.3	1/32	3	3/22	14
	Body pain	191/429	45	64/146	44	1/5	20	4/21	19	6/12	50
	Chills	169/526	32	52/159	33	6/12	50	5/31	16	4/20	20
	Malaise	150/464	32	45/147	31	3/10	30	5/26	19	3/14	21
	Headache	346/452	77	115/153	75	5/10	50	12/23	52	9/11	82
**Signs and symptoms cont.**											
	Vomiting	96/586	16	17/168	10	6/17	35	2/33	6	4/24	17
	Diarrhea	39/586	7	10/168	6	0/17	0	4/33	12	2/24	8
	Abdominal pain	100/528	19	25/164	15	5/17	29	6/27	22	4/18	22
	Ear pain	24/552	4	6/166	4	0/17	0	0/31	0	0/21	0
**Influenza vaccination history**											
**Yes**		63	12	20	14	6	60	10	44	3	14
**No**		520		148		10		23		21	
**Previous treatment**											
	Antipyretics	384	66	113	67	11	65	22	67	15	63
	Antibiotics	133	23	34	20	6	35	7	21	5	21
** **	Antivirals	0	0	0	0	0	0	0		0	0

* children <1 yr were excluded

** The Battambang sentinel site consists of 2 health centers (Thmor Koul and Tapoung) within close proximity and serve the same community.

Influenza vaccination coverage in 2010 and 2011 approximated 20% in response to the influenza A/pH1N1(2009) pandemic yet decreased to zero in 2012 by subjects’ reports. Five hundred and seventeen ILI cases were taking medication at the time of presentation: 384 (66%) antipyretics (paracetamol) and 133 (23%) antibiotics. Antibiotic use was higher than average (35%) in patients positive for enterovirus. No use of antivirals was reported.

**Fig 3:**
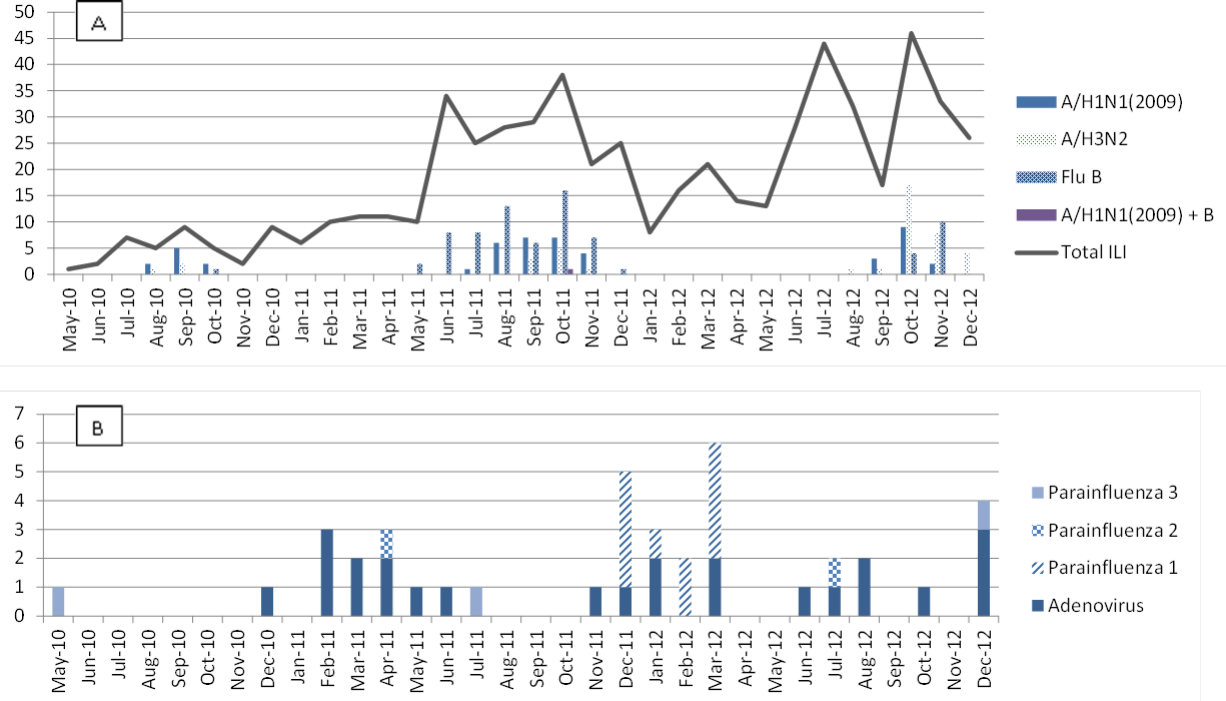
Influenza and other respiratory viruses detected between 2010–2012. (A) Number of ILI and influenza subtypes, May 2010-Dec 2012. (B) Number of other respiratory viruses among influenza-negative ILI specimens May 2010-Dec 2012.

Between 2010 and 2012, influenza cases in Western Cambodia were almost exclusively seen in the rainy season (June to November), with almost no influenza detected in the dry seasons (December to May). Dominant influenza subtypes were A/pH1N1(2009) in 2010, influenza B in 2011 and influenza A/H3N2 in 2012. No human influenza A/H5N1 cases were detected. Antigenic analysis showed that all A/pH1N1 influenza strains found in 2011 (n = 13) belonged to the A/California/7/2009-like group and matched the reference strains included in the southern hemisphere vaccine for 2011. The four A/H3N2 influenza strains in 2011 all belonged to the A/Perth/16/2009-like strain. The B influenza strains belonged to either the B/Brisbane/60/2008-like group (n = 20) or the B/Malaysia/2506/2004-like group (n = 5) but all were within the Victoria lineage of influenza B, showing only a partial matching with the reference strain in the 2011 vaccine (Table 3 and Figs 2 and 3A). Vaccination coverage during the entire study period was 11% (63/583). The probability of developing influenza among ILI-patients who had been vaccinated (20/63) and who had not (148/520) was similar and not significantly different (0.32 vs. 0.28, *p* = 0.28). All IC_50_-values of influenza A and B isolates tested from 2011 were within the susceptible range for both oseltamivir and zanamivir (Table 3).

**Table 3:** IC_50_-values (nM) of influenza A and B isolates from 2011 to oseltamivir and zanamivir [Range(s) = Range for sensitive control isolates; Range(r) = Range for resistant control isolates).

Drug	Strain	IC50 (nM)-NA-Star					IC50 (nM)-MUNANA			
		n	Range	Mean ± SD	Range (s)^a^	Range (r)^b^	n	Range	Mean ± SD	Range (s)
**Oseltamivir**	A/California/7/2009	7	0.35–0.86	0.58 ± 0.18	0.2–10.0	101.9–195.9				
**Oseltamivir**	B/Brisbane/60/2008	8	0.87–8.13	4.74 ± 2.28	0.2–11.6		5	5.89–19.8	12.7 ± 5.39	0.1–40.3
**Oseltamivir**	B/Malaysia/2506/2004						4	1.43–17.3	9.18 ± 6.99	
**Zanamivir**	A/California/7/2009	7	0.56–1.52	0.78 ± 0.33	0.1–13.5	1.5–1.9				
**Zanamivir**	B/Brisbane/60/2008	8	0.61–14.4	10.14 ± 4.33	0.4–17.9		5	1.16–2.67	1.80 ± 0.58	0.1–4.1
**Zanamivir**	B/Malaysia/2506/2004						4	1.67–3.00	2.41 ± 0.57	

^a^ IC_50_ range (nM)for sensitive wild type influenza A/H1N1 and influenza B isolates.

^b^ IC_50_ range (nM) for resistant control A/H1N1 isolate A/Mississippi/274Y

### Other respiratory viruses

Overall, at least 1 respiratory virus was detected in 258 out of 586 (44%) ILI-specimens collected between May 2010 and December 2012 (Fig 2); of which most were influenza (168, 29% of ILI-cases), followed by rhinovirus (33, 6%), adenovirus (24, 4%), non-polio enterovirus (17, 3%) and parainfluenza virus (PIV) (16, 3%). All of the 418 ILI specimens that tested negative for influenza A or B by rRT-PCR were sent for viral culture. Forty (10%) were culture positive; 24 adenovirus, 11 PIV1, 2 PIV2, 3 PIV3 were isolated (Figs 2 and 3B). No RSV, HMPV or coronaviruses were identified.

**Fig 4:**
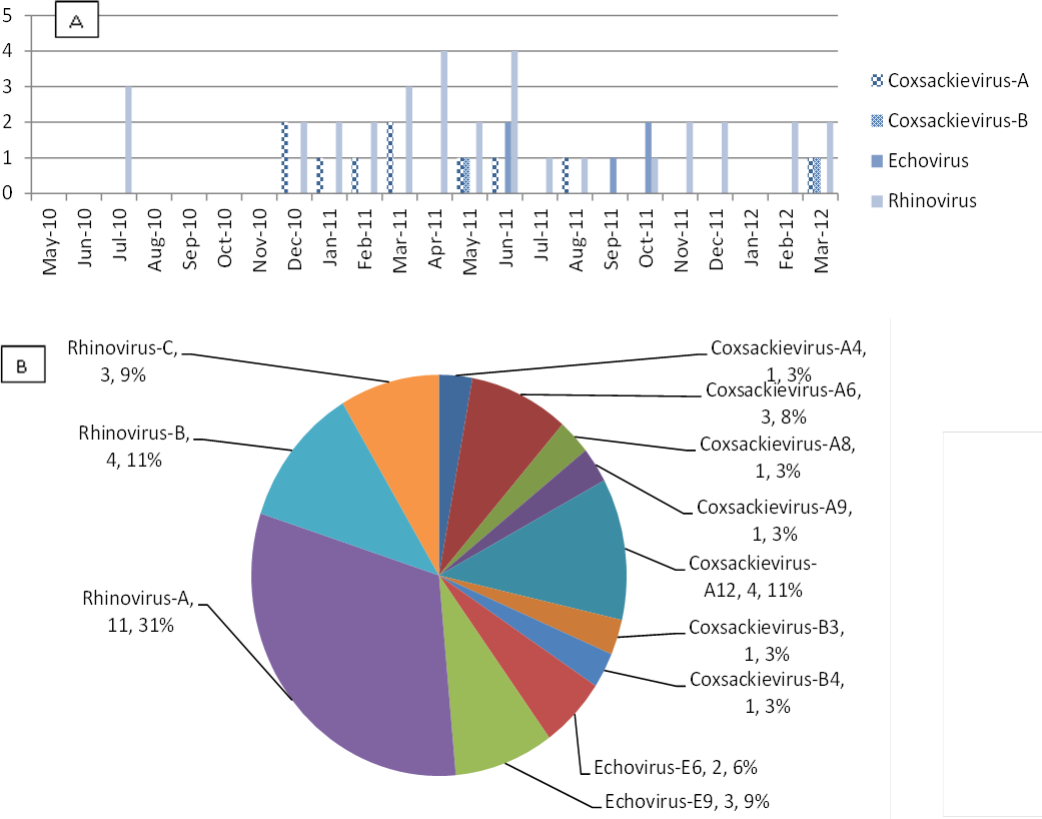
Enteroviruses and rhinoviruses detected by viral culture and sequencing between May 2010-April 2012. (A) Number of enteroviruses and rhinoviruses detected by EV/RV assay in 164 culture negative specimens, May 2010-April 2012. (B) Number and percentage of enterovirus and rhinovirus serotypes among a total of 50 (17 EV and 33 RV) positive samples.

Pan-EV/RV and EV71 nested RT-PCR (S4 Table) detected 17 samples positive for enterovirus, which included coxsackievirus A (n = 10, 1 A4, 3 A6, 1 A8, 1 A9, 4 A12), echovirus (n = 5, 2 E6, 3 E9) and coxsackievirus B (n = 2, B3, B4). A single specimen was found to be negative during the first round of RT-PCR but positive by second round pan-EV/RV and EV71 nested RT-PCR. The 662 base pairs PCR product fragment obtained from pan-EV/RV nested RT-PCR was sequenced using the RV/EV inner primers. The 528 bases obtained from sequencing were found to be 99% identical to the Enterovirus A71/Homo sapiens/VNM/208/2011 strain (Accession: KJ686294). When the 227 bp PCR product obtained from EV71 nested RT-PCR was sequenced using the EV71 inner primers, 131 bases was obtained. This sequence was found to be 99% identical to Enterovirus A71/Cambodia: Banteay Meanchey 2012 strains (Accessions: KP308459, KP308453, KP308450, KP308448, KP308430, KP308427, KP308410, KP308406). Out of the 33 specimens positive with rhinoviruses, only 18 could be serotyped. Of these 18, most were rhinovirus A (n = 11), followed by rhinovirus B (n = 4) and rhinovirus C (n = 3) (Fig 4B). Amplicons were sequenced and sequences were analyzed using Genbank’s BLAST tool to identify the virus species.

### Phylogenetics

#### Pandemic influenza A (pH1N1)

**Fig 5:**
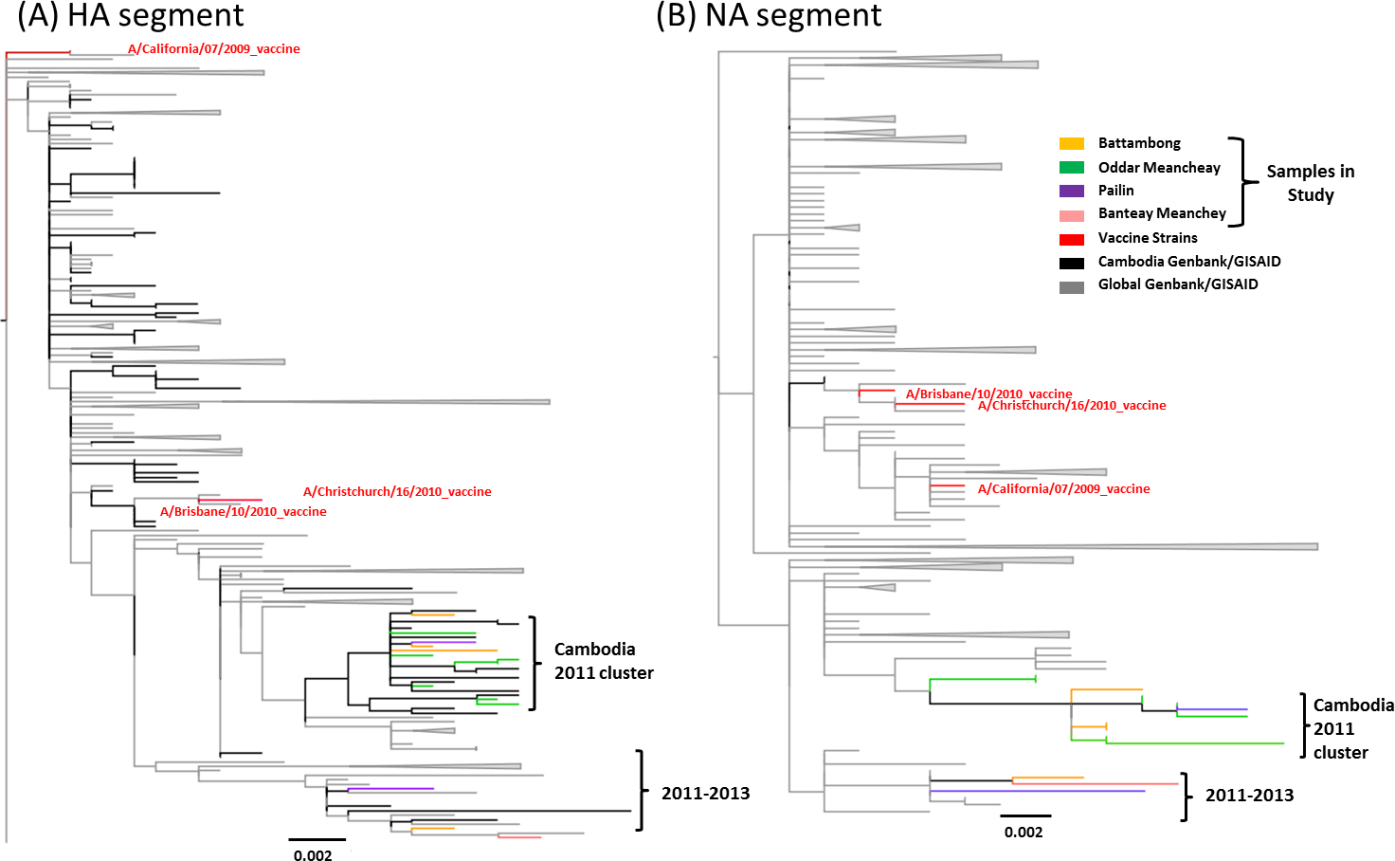
**pH1N1 maximum likelihood phylogenetic trees** (aLRT support >70 for all vaccine clades and major nodes) for the (A) HA and (B) NA segments for 14 samples from Cambodia and references in Genbank (grey) collected in 2011 and 2012. Vaccine strains are highlighted in red. Samples are colored coded by sampling site.

All sequences were highly related with no more than 1.67% divergence between the strains detected in the study and respective seed viruses included in the vaccine composition (A/California/7/2009, A/Brisbane/10/2010, and A/Christchurch/16/2010; S5 Table, Fig 5) for any of the segments analyzed. When compared to the A/California/7/2009 vaccine strain, all Cambodian viruses on average had 11 amino acid changes within the HA segment. Amino acid changes found in the HA gene can be found in Table 4. No evidence for amino acid substitutions leading to changes in glycosylation was seen in the HA dataset; however, several changes among sequenced samples occurred in antigenic sites, polymorphic sites or had a recorded effect in *in vitro* analyses per the literature (S6 Table). For the NA gene, several amino acid substitutions resulted in addition/loss of glycosylation sites, or were found in polymorphic or antigenic sites (epitopes) (S6 Table). For the MP gene, all samples contained the S31N mutation that may confer amantadine resistance as determined by the Genbank Influenza Viral Resource Annotation tool [32]. No features or changes of interest were seen within the NP or NS segment. The HA and NA segments contained the most amino acid substitutions when compared to the reference (A/California/7/2009) as well as to the yearly defined groups (2011 and 2012). The MP and NP segments showed the fewest substitutions (Table 4). There were no samples within the HA amino acid analysis that had unique substitutions relative to the global sequences. However, the NA gene had unique amino acid substitutions not found with the global references; the function of these substitutions is unknown (S7 Table). Selection pressure was determined using the dN/dS statistic. Values of dN/dS > 1 are indicative of positive or adaptive selection, values <1 are indicative of purifying or negative selection and a value = 1 indicates neutral or no selection ongoing in the dataset. All samples were under purifying selection for all segments with exception to the NS segment of the 2011 Cambodian samples, which had a dN/dS value of 1.0 suggesting neutral selection (S8 Table).

**Table 4:** pH1N1 and H3N2 amino acid changes ^a^ specific to sample sets for the HA and NA genes.

Influenza Subtype	Segment	Sample sets (n) and AA changes found										
(vaccine compared to)		All ^d^	2011^d^	2012^d^	Individual samples (n)							
					BTB		OM		PL		BMC	
					2011 (5)	2012 (3)	2011 (8)	2012 (5)	2011 (2)	2012 (1)	2011 (0)	2012 (1)
**pH1N1 (A/California/7/2009) n = 15**	HA	E391K	N277D/G	H155R	G172E (1)	G172E (1)	N173D (1)		G172E (1)			G172E(1), S200P (1)
**pH1N1 (A/California/7/2009) n = 15**	NA	N248D	N44S	D451G		N63S(1)						I396V(1)
**H3N2 (A/Victoria/361/2011) n = 10**	HA	Q172H, V202G, Y235S		N161S	N24D (2)	N61S (1) I64T (1) D69S (1) A154S (1) S225R (1)	I156M (1) N161S (1)	N61S (4) I64T (4) D69S (4) A154S (1) T147K (1)	N24D (1)			
**H3N2 (A/Victoria/361/2011) n = 10**	NA	K258E, T329N					G401S (1)	D151N (1), D402N (1)				

BTB: Battambang, OM: Oddar Meanchey, PL: Pailin, BMC: Banteay Meanchey

^a^ All substitutions were also found within global sequences. Only those substitutions noted in previous studies are included.

For pH1N1 there was no evidence of geographic clustering of sequences within sampling site (colored branches in Fig 5); nor was there evidence of reassortment among the sequences sampled. Sequences (references and samples) from Cambodia 2011 distributed throughout the trees; however, the majority of 2011 sequences, including those corresponding to strains detected in this study appeared within a 2011 specific Cambodian clade. However, this clade was only evident in HA and NA phylogenies and did not appear in NP, MP or NS phylogenies (S1–S5 Figs).

#### Influenza A H3N2 (H3N2)

**Fig 6:**
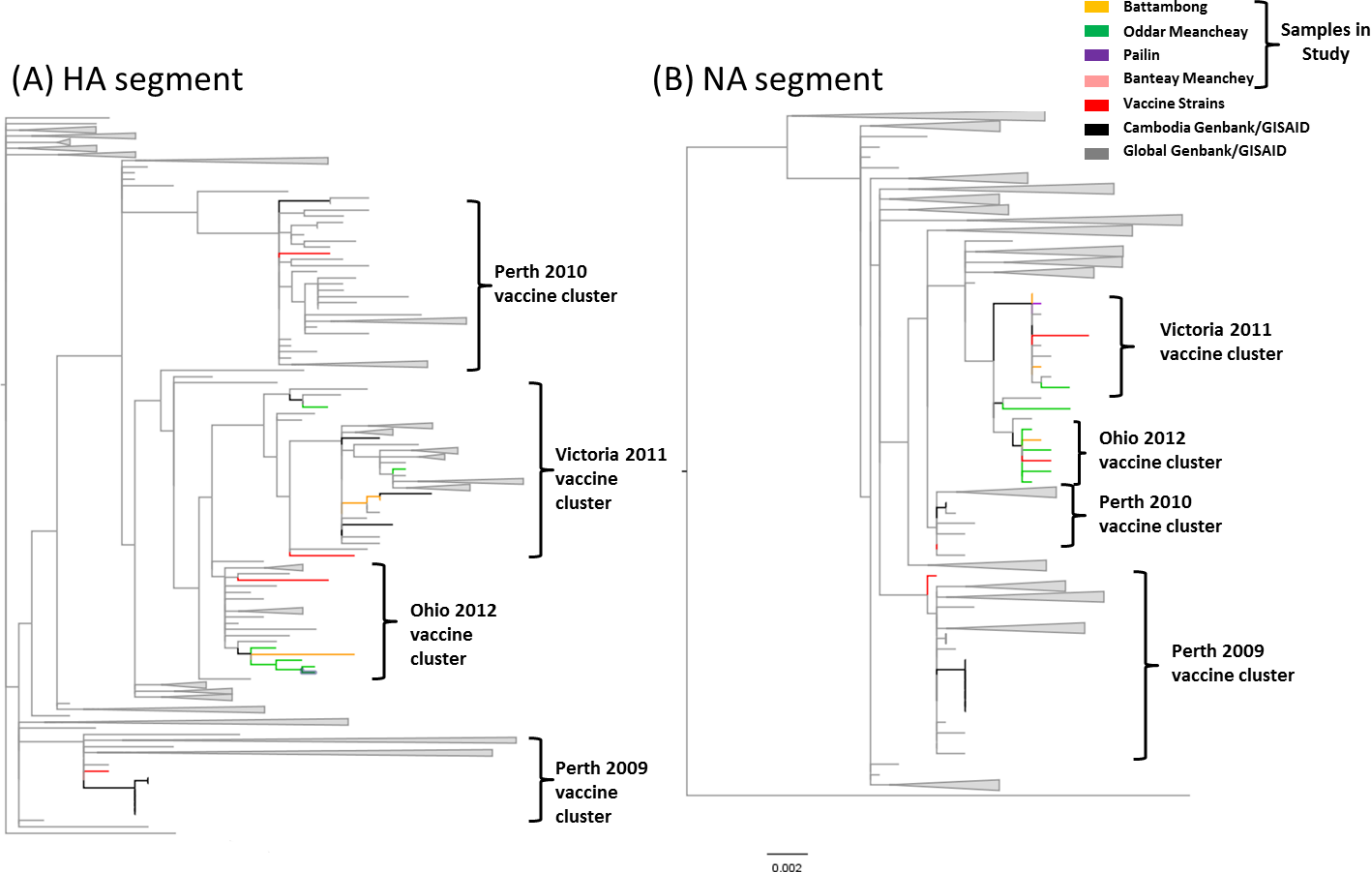
**H3N2 maximum likelihood phylogenetic trees** (aLRT support >70 for all vaccine clades and major nodes) for the (A) HA and (B) NA segments for 10 samples from Cambodia and references in Genbank (grey) collected in 2011 and 2012. Vaccine strains are highlighted in red. Samples are colored coded by sampling site.

All sequences were highly related with no more than 1.77% divergence between the samples and the furthest related respective vaccine (S9 Table, Fig 6) for any of the segment. The majority of segments were more closely related to the A/Victoria/361/2011 vaccine strain; therefore all subsequent analyses were compared to this strain. For the HA gene, when compared to the A/Victoria/361/2011 vaccine strain, all samples on average had 9 amino acid changes within the HA segment (S10 Table). There were several amino acid changes among the sequenced samples occurring in antigenic sites, polymorphic sites or which resulted in a change of glycosylation (Table 5). For the NA gene, several amino acid substitutions resulted in addition/loss of glycosylation sites, or were found in polymorphic or antigenic/catalytic sites (epitopes) (Table 5). For the MP gene, it was noted that all samples contained the S31N mutation that may confer adamantane resistance as determined by the Genbank Influenza Viral Resource Annotation tool [32]. No features or changes of interest were seen within the NS segment for any sample.

**Table 5:** Changes in glycosylation, antigenic and polymorphic sites linked to amino acid (AA) substitutions and sub-antigenic/catalytic sites (epitopes) where AA substitutions were found for hemagglutinin (HA) and neuraminidase (NA) segments of H3N2.

Segment	AA Substitution^a^	Notation
HA^b^	N161S	Antigenic region A [33–35]
HA^b^	Q172H	Antigenic region B [33–35]
HA^b^	V202G	Antigenic region B [33,34]
HA^b^	Y235S	Antigenic region D [33–35]
HA^b^	N61S	Loss of glycosylation site (NSS to SSS); Antigenic region C [33,34]
HA^b^	I64T	Antigenic region C [33,34]
HA^b^	D69S	Antigenic region C [33,34]
HA^b^	A154S	Antigenic region A [33,34]
HA^b^	S225R	Antigenic region D [33–35]
HA^b^	T147K	Antigenic region A [33,34]
HA^b^	N24D	Loss of glycosylation site (NST to DST)
HA^b^	I156M	Antigenic region A [33–35]
NA^c^	K258E	Unknown
NA^c^	T329N	Addition of glycosylation site (TDS to NDS)
NA^c^	D151N	Catalytic site [35]
NA^c^	G401S	Antigenic site A
NA^c^	D402N	Addition of glycosylation site (DRS to NRS)

AA: amino acid

^a^ Amino acid of reference on left, sample substitution on right of amino acid position number.

^b^ HA numbering starts from Methionine as position 1.

^c^ NA numbering starts from Methionine as position 1.

The HA and NA segments contained the most amino acid substitutions when compared to the reference (A/Victoria/361/2011) as well as to the yearly defined groups (2011 and 2012). The MP and NS segments showed the fewest substitutions (S10 Table). There were several amino acid substitution within the HA amino acid analysis that were unique relative to the global sequences (and vaccine references); two of which occurred in antigenic regions (Table 5) and five additional changes of unknown effect or location significance (S11 Table). One amino acid substitution in the NA gene was found to be unique in antigenic site A (Table 5) and 7 unique substitutions of unknown effect or location significance were also found (S12 Table). All ‘unique’ AA substitutions were not found with the global sequences or vaccine references. All samples were under purifying selection for all segments with the exception to the NS segment. When samples were separated based on the respective years of collection, the NS gene showed evidence of neutral selection (dN/dS ~ 1). However, when all samples were combined to increase sample size, it showed overall evidence of purifying selection (dN/dS < 1; S10 Table).

**Fig 7:**
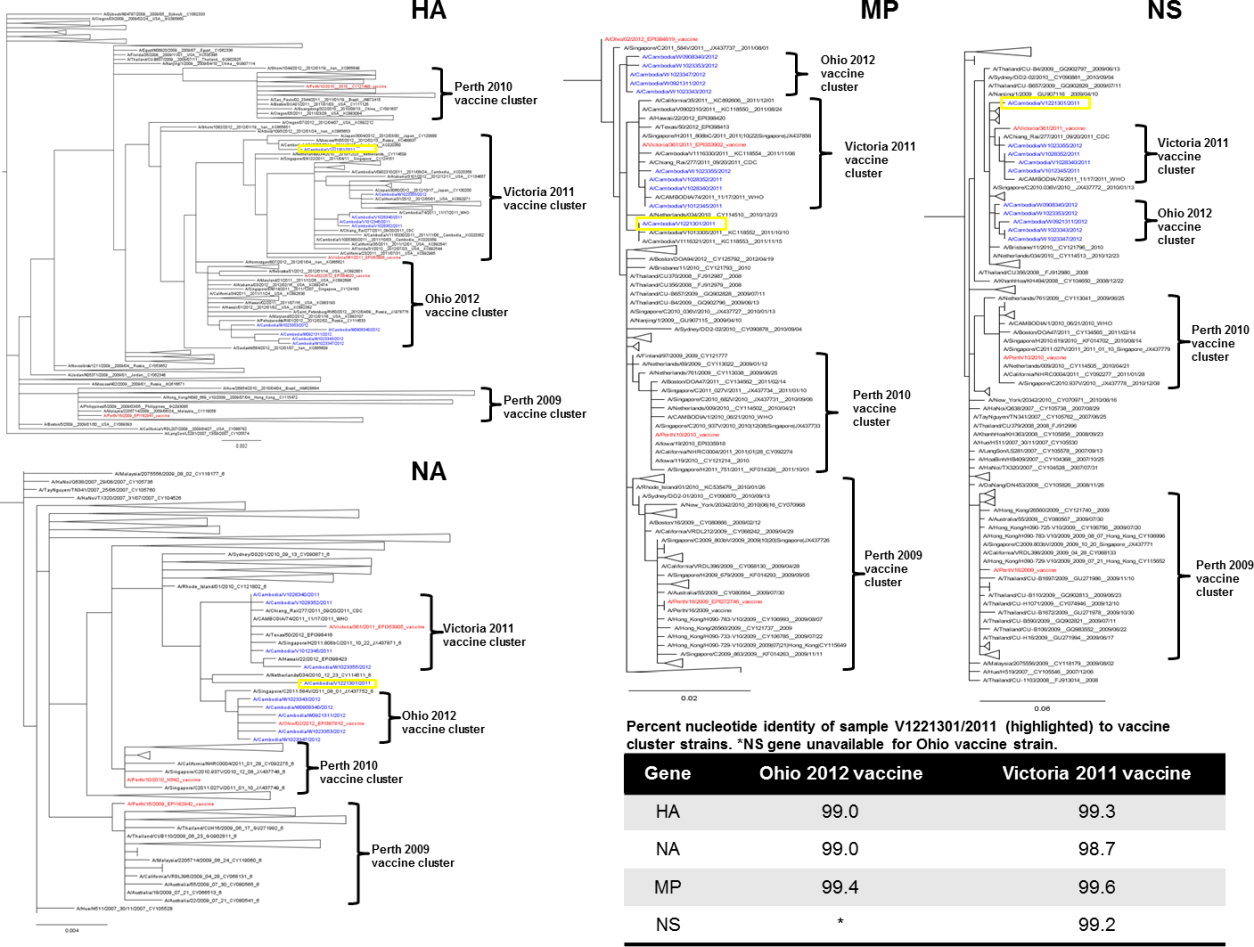
H3N2 maximum likelihood phylogenetic trees (aLRT support >70 for all vaccine clades and major nodes) for all segments analyzed in this study for 10 samples from Cambodia (colored in blue) and references in Genbank collected in 2011 and 2012. Vaccine strains are highlighted in red and clades defined by brackets. Unique sample that did not fall within vaccine clades highlighted in yellow with percent nucleotide identity indicated in bottom right table.

Overall, for H3N2, there was no evidence of geographic clustering of sequences within the same sampling site except samples from Oddar Meanchey in which all but one sample clustered into the Ohio 2012 vaccine cluster (green branches in Fig 6). This clustering was visual only, not statistically significant due to small sample size, though the cluster was well supported (cluster node value 81, value obtained using associated likelihood ratio test (aLRT); analogous to bootstrap analysis). There was no evidence of reassortment among the samples analyzed and all samples were consistently grouping with either the A/Ohio/02/2012 or A/Victoria/361/2011 vaccines. There was one exception; sample A/Cambodia/V1221301/2011 consistently fell outside of both the Ohio 2012 and Victoria 2011 vaccine clusters (Fig 7) for all segments analyzed.

## Discussion

This is to the best of our knowledge the first influenza surveillance study in remote border areas in western Cambodia. Over the time course of 31 months, spanning 3 influenza seasons including the last few months of pandemic A/pH1N1(2009) influenza circulation, seasonal influenza virus was the most commonly detected respiratory virus in this predominantly pediatric population of subjects who presented with ILI (Fig 3).

Despite detection of human highly pathogenic avian influenza (HPAI) H5N1 virus in Western Cambodia since early 2011 [36–38], H5N1, typically detected in south-central Cambodia, was not found at our sentinel sites. Influenza subtypes varied by year with A/pH1N1(2009) being predominate in 2010, influenza B in 2011, and influenza A/H3N2 in 2012. We did not expect to detect any H5N1 in our laboratory surveillance system as most suspected H5N1 cases would have been screened out by the health centers based on reported exposure to dead or diseased poultry. The specimen of one patient with reported H5N1 exposure in 2011 was investigated in our laboratory and tested negative for H5N1.

No national policies have been established in Cambodia for seasonal influenza vaccination to date. History of vaccination was low in this population. Available influenza vaccines during the study period were the A(H1N1)2009pdm monovalent vaccine in the public sector and the trivalent seasonal vaccine in 2009, 2010 and 2011 in the private sector [39]. Our data revealed that the influenza B did not match well with strains included in vaccine composition of trivalent vaccines; therefore, if vaccination is to be implemented, the quadrivalent influenza vaccine that contains the two influenza B lineages, rather than the trivalent vaccine, may be more suitable. Aside from a limited quantity of oseltamivir, stockpiled by the government for management of severe disease by pandemic or avian influenza [40], use of antivirals for influenza is very low in Cambodia [40]. As a result, all strains we detected were susceptible to neuraminidase inhibitors (NIs) such as oseltamivir and zanamivir, which can be partially explained by the absence of these medications in Cambodia. Due to widespread adamantane resistance, we did not conduct susceptibility testing on this class of antiviral drugs; however, we did find the S31N mutation conferring adamantane resistance in all of our influenza isolates tested.

Phylogenetically, all our samples clustered together within each of the two subtypes (A/pH1N1 or A/H3N2), which is not unusual given the location and specimen collection dates compared with the reference sequences used. Our western Cambodian pH1N1(2009) isolates were more closely related, based on full genome analysis, to 10 earlier isolates from Cambodia (94.4% genome conservation) than to the 13 Thai isolates (75.9% genome conservation) or the California 2009-vaccine reference. However, it was also noted that amino acid changes were shared with the Thai references suggesting a possibility of mixing between Thai and Cambodian influenza as expected. Isolates from Battambang, being western Cambodia's major transportation hub, showed the highest diversity of amino acid changes.

Analysis of sequence data from our influenza patients revealed a random or neutral mutation of the influenza genomes as expected. Koel et al. [41] discovered that periodic major antigenic change in influenza A/H3N2 virus was caused mainly by single amino acid substitutions, which occurred at only seven out of 131 possible amino acid positions in HA at antigenic sites immediately adjacent to the receptor binding site (RBS). These substitutions were located in antigenic sites A (position 145) and B (positions 155, 156, 158, 159, 189, and 193), with none in sites C, D, or E. We did not see these changes within antigenic sites A or B in our dataset.

The HA and NA genes in our H1 and H3 isolates showed signatures of purifying selection meaning that the virus keeps these genes conserved by removing random mutations as they occur. These single amino acid mutations were found at non-epitope sites of HA [41] that have not been associated with major antigenic changes on their own. However, it is possible that some of these mutations may constitute permissive or compensatory mutations that would be important in enabling co-mutations that could affect viral fitness, and as such, have an incentive to remain fixed under purifying selection. Permissive mutations have been described in the spread of oseltamivir resistant influenza A/H1N1 virus that carry the H274Y mutation and are increasingly being recognized as a major force in evolution [42,43].

Glycosylation (addition of oligosaccharide chain to the surface protein) is another common form of protein modification. Alteration of glycosylation sites can affect folding and conformation changes in the surface glycoprotein, hereby impacting virus survival and transmissibility. In addition, glycosylation can affect interaction with receptors and cause a virus to be more [or less] recognizable by the innate host immune system and antibodies [44]. None of the changes in glycosylation in our isolates have been reported in the literature as having an effect on viral structure or function; but more data may be needed. Mapping sites of mutation and glycosylation on epitopes provides a better understanding of antigenic drift and is important for improving vaccine strategies [45].

Overall, and despite adequate storage and transportation of our specimens, culture yield was low in our dataset (~10%) with isolation of only adenovirus and parainfluenza. This may be due to an exclusion of children younger than 1 year, the outpatient setting with less severe disease and possibly a later presentation to the health facility (which was possible up to 5 days after fever onset) resulting in a lower viremia. Previous work among hospitalized patients has shown presence of human coronavirus, human bocavirus, HMPV and RSV in Cambodia [2] but these were not detected by our culture techniques. Therefore, the number of non-influenza viruses in our population is possibly underestimated. Additional testing is ongoing at AFRIMS in order to elucidate true disease burden of non-influenza viruses on the population.

In comparison with previous work in Cambodia [46], we found a relatively higher proportion (9.3% versus 1.3%) of ILI-patients testing positive for adenovirus over 2 dry seasons in 2011 and 2012 which may be explained by our younger study population who may be disproportionately affected by this virus. PIV-3 (0.9%), which in previous dry seasons was described as the most common parainfluenza-virus in Cambodia [2], was the least common type during our surveillance period, whereas PIV-1 (5.6%) was most common, particularly during November 2011 to March 2012. This is not consistent with previous reports for Cambodia [46]. The lack of presence of PIV-3 in our study population may be due to an outbreak of PIV-1 during the surveillance time frame and the fact that we did not include children under 1 year old, an age when 40% of PIV-3 is usually detected [47].

By employing a highly sensitive and specific nested RT-PCR assay for enteroviruses and rhinoviruses, we found a higher proportion (5.6%) of non-polio enteroviruses in children less than 5 years old compared with previous studies (1%) in Cambodia [2] which could be partially explained by the predominantly outpatient population in our study sample. This is likely an underestimation of the true prevalence in this population since we excluded children less than one year of age, an age group more likely infected by enteroviruses at rates exceeding those of older children and adults by several fold [48–50]. The most common enteroviruses found among children under 5 years old in our population were coxsackieviruses (7.3%) and echoviruses (3%). Although we did not find coxsackievirus A16, the most common etiologic agent for hand-foot-mouth-disease (HFMD), we found other coxsackieviruses that can cause HFMD such as coxsackievirus A6 (n = 3) and A12 (n = 4). Coxsackievirus A6 is known to cause either an atypical rash resembling eczema herpeticum or chickenpox (United States 2011–2012 and in Europe since 2009) or nail loss one to two months after onset of symptoms (Finland 2009, Taiwan 2010, Japan 2011). Coxsackievirus A12 was one of the enteroviruses implicated in HFMD outbreaks in China in 2008 and 2009 [51]. While these patients reported symptoms of fever (38.1–39°C), runny nose, chills, cough, congestion and abdominal pain, they did not report symptoms specific to HFMD such as herpangina and skin rash.

We did not detect in our study EV71 virus genotype C4, which was the principal etiologic agent causing the 2012 HFMD outbreak across Southeast-Asia that manifested as a life-threatening neuro-respiratory syndrome in 78 children under 3 years old from 14 provinces in Cambodia [52]. Although we did not have EV71 test results from specimens collected during the peak of the outbreak, our data suggests that EV71 virus was not present yet in the border areas of Western Cambodia before April 2012, the time that the first cases were found elsewhere in Cambodia.

Regarding testing for non-influenza viruses, the use of the less sensitive viral culture as the first-line test supplemented by IFA on only a subset of influenza-negative ILI-specimens underestimates overall extent of transmission as well as co-infections. Exclusion of children under 1 year of age may also contribute to underestimation. Our data was derived from a passive surveillance system with convenience sampling. While we attempted to select surveillance sites representative of the nearby geographic locations, we had no accurate figures of the catchment populations or denominators at our respective sentinel sites. Our staggered method of adding sentinel sites suggests that not all sentinel sites enrolled patients evenly over 3 influenza seasons. Similarly, with a small sample size and limited number of sampling locations, no inferences could be made with regard to temporal or spatial flow of influenza strains in our phylogenetic analysis. Now with a more established system in place, our data may provide a useful baseline for future molecular evolution studies of influenza in Cambodia and in the region.

## Conclusions

Despite proximity to Thailand, influenza activity, seasonality, antigenicity and anti-viral susceptibility in western Cambodian isolates followed patterns observed elsewhere in Cambodia rather than Thailand. This supports earlier recommendations from the Cambodian NIC to use the northern hemisphere influenza vaccine on a southern hemisphere vaccination schedule. Additionally, use of the quadrivalent versus the trivalent vaccine should improve coverage of influenza B strains circulating in Cambodia. Neuraminidase inhibitors may still be used for treatment and chemoprophylaxis for seasonal influenza given little evidence for resistance. The amino acid mutations at non-epitope sites, in particular of HA, require further investigation in light of the increasingly important role of permissive mutations in the evolution of influenza virus. Further research to clarify the burden of adenovirus and non-polio enteroviruses as etiologic agents in acute respiratory infections in Cambodia is needed.

## Supporting Information

S1 Fig pH1N1 maximum likelihood phylogenetic trees for the HA segment for 14 samples from Cambodia (colored in blue) and references in Genbank (colored in black) collected in 2011 and 2012 (also see main text Fig 1A). Vaccine strains are highlighted in red. Node support was calculated with aLRT and was >0.70 for all major nodes. The 2011 samples fell within the same clade and 2012 samples fell within a different clade, all samples clustered with other sequences isolated from Cambodia.
(TIF)

S2 Fig pH1N1 maximum likelihood phylogenetic trees for the MP segment for 15 samples from Cambodia (colored in blue) and references in Genbank (colored in black) collected in 2011 and 2012. Vaccine strains are highlighted in red. Node support was calculated with aLRT and was >0.70 for all major nodes. All samples fell within the same major clade that included other sequences isolated from Cambodia.
(TIF)

S3 Fig pH1N1 maximum likelihood phylogenetic trees for the NA segment for 14 samples from Cambodia (colored in blue) and references in Genbank (colored in black) collected in 2011 and 2012 (also see main text Fig 1B). Vaccine strains are highlighted in red. Node support was calculated with aLRT and was >0.70 for all major nodes. The 2011 samples fell within the same clade and 2012 samples fell within a different clade.
(TIF)

S4 Fig pH1N1 maximum likelihood phylogenetic trees for the NP segment for 15 samples from Cambodia (colored in blue) and references in Genbank (colored in black) collected in 2011 and 2012. Vaccine strains are highlighted in red. Node support was calculated with aLRT and was >0.70 for all major nodes. The 2011 samples fell within the same clade and 2012 samples fell within a different clade.
(TIF)

S5 Fig pH1N1 maximum likelihood phylogenetic trees for the NS segment for 15 samples from Cambodia (colored in blue) and references in Genbank (colored in black) collected in 2011 and 2012. Vaccine strains are highlighted in red. Node support was calculated with aLRT and was >0.70 for all major nodes. The 2011 samples fell within the same clade and 2012 samples fell within a different clade.
(TIF)

S1 Table Forward and reverse primers used in amplification of influenza viruses with 5' and 3' modifications indicated. (DOCX)

S2 Table Samples collected for genetic analysis in this study including virus subtype, sample origin, specimen type, sampling date and segments successfully sequenced. (DOCX)

S3 Table Primers for RT-PCR and nucleotide sequencing for EV/RV and EV71: The enterovirus 71 type specific primers were modified from the previous study by Singh et al. (2002) [53]. The modifications were made by following the alignments of VP1 sequences of different EV71 strains collected during 2002–2011 from Thailand, Taiwan, Philippines, Vietnam, and China available in Genbank including the sequences with the accession No. JN191177-9, FJ969151, FJ969163, JQ 621835, JQ621841, AM490141-63, JQ315092, and JX203305. The primers for simultaneous detection of enteroviruses and rhinoviruses were designed in the polyprotein gene, between 5′ non-coding region (5′NCR) and VP4/VP2 regions that was previously described by Coiras et al. 2004 [29]. EV/RV-2n was modified from primer 2-EV/RV [29] for using in nested PCR reaction.
(DOCX)

S4 Table Total number of viruses identified by pan-enteroviruses and rhinoviruses (EV/RV) PCR and nucleotide sequencing^†^ (DOCX)

S5 Table pH1N1 average percent nucleotide sequence identity between vaccine strains and Cambodia isolates gene segments. (DOCX)

S6 Table Changes in glycosylation, antigenic and polymorphic sites linked to AA substitutions and sub-antigenic/catalytic sites (epitopes) where amino acid (AA) substitutions were found for for the HA and NA segments of pH1N1. AA substitution nomenclature is as follows; reference amino acid (A/California/7/2009), amino acid site, sample amino acid. Amino acids are numbered from the start codon of the segment (ATG:Methionine).
(DOCX)

S7 Table Unique pH1N1 amino acid (AA) changes of unknown function in specific samples for the NA gene as compared to A/California/7/2009. AA substitution nomenclature is as follows; reference amino acid (A/California/7/2009), amino acid site, sample amino acid. Amino acids are numbered from the start codon of the segment (ATG:Methionine).
(DOCX)

S8 Table pH1N1 sample amino acid substitution summary and selection analysis by group per segment analyzed. (DOCX)

S9 Table H3N2 average percent nucleotide sequence identity between vaccine strains and Cambodia isolates gene segments. (DOCX)

S10 Table H3N2 sample amino acid substitution summary and selection analysis by group per segment analyzed. (DOCX)

S11 Table Unique H3N2 amino acid changes of unknown function to specific to samples for the HA gene as compared to A/Victoria/361/2011. AA substitution nomenclature is as follows; reference amino acid (A/Victoria/361/2011), amino acid site, sample amino acid. Amino acids are numbered from the start codon of the segment (ATG:Methionine).
(DOCX)

S12 Table Unique H3N2 amino acid changes of unknown function to specific to samples for the NA gene as compared to A/Victoria/361/2011. AA substitution nomenclature is as follows; reference amino acid (A/Victoria/361/2011), amino acid site, sample amino acid. Amino acids are numbered from the start codon of the segment (ATG:Methionine).
(DOCX)

S13 Table Sequencing results of the EV71 isolates based on (A) pan rhinovirus/enterovirus inner primer and (B) Sequencing results of the EV71 isolates based on an enterovirus 71 inner primer.
(DOCX)
