# Supplementary figures and images for: Human Sentinel Surveillance of Influenza and Other Respiratory Viral Pathogens in Border Areas of Western Cambodia
Source: PLoS One. 2016 Mar 30;11(3):e0152529. doi: 10.1371/journal.pone.0152529 (PMC4814059; doi:10.1371/journal.pone.0152529)

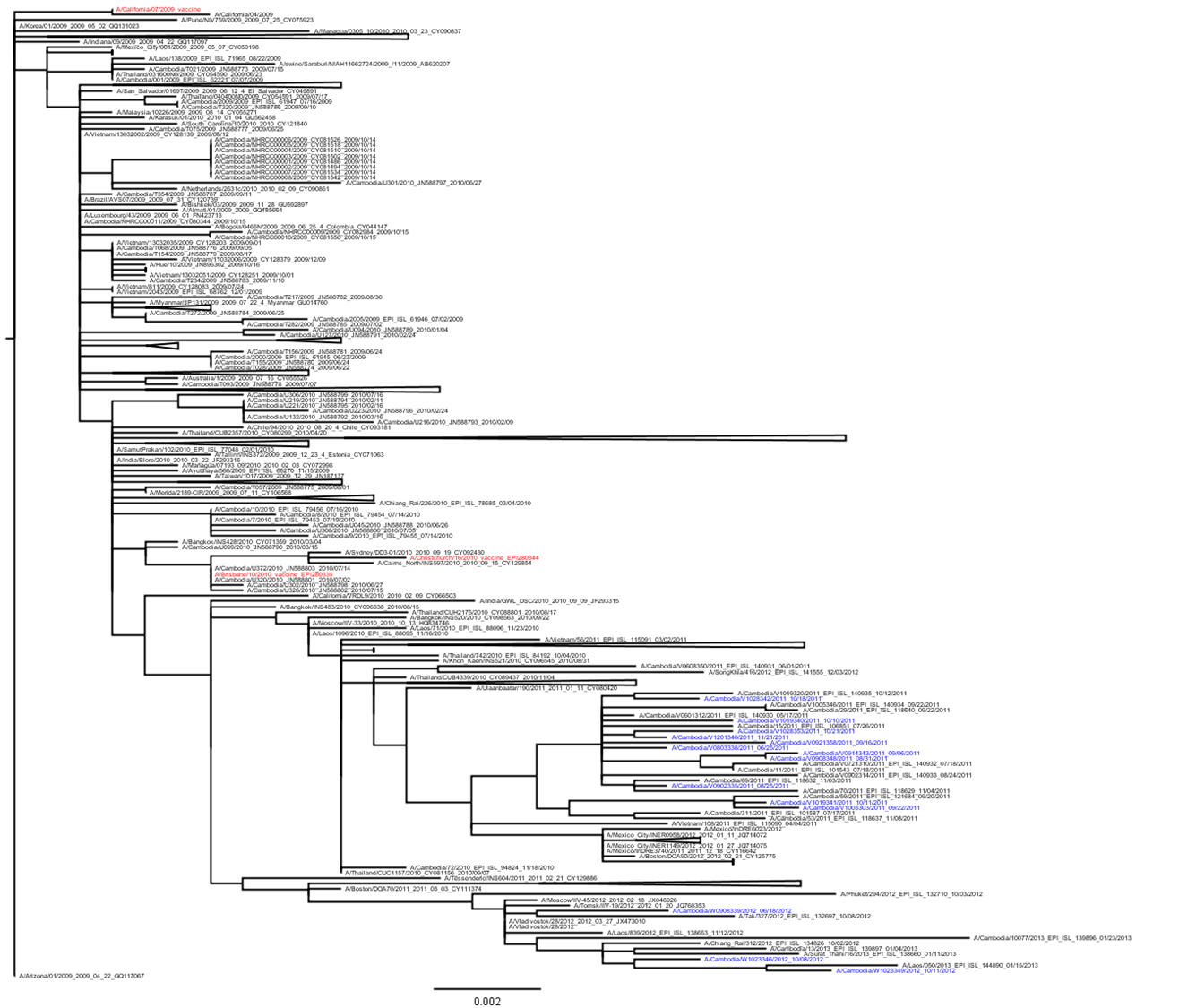

Supplement: S1 Fig — Vaccine strains are highlighted in red. Node support was calculated with aLRT and was >0.70 for all major nodes. The 2011 samples fell within the same clade and 2012 samples fell within a different clade, all samples clustered with other sequences isolated from Cambodia. (TIF) [file pone.0152529.s001.tif]

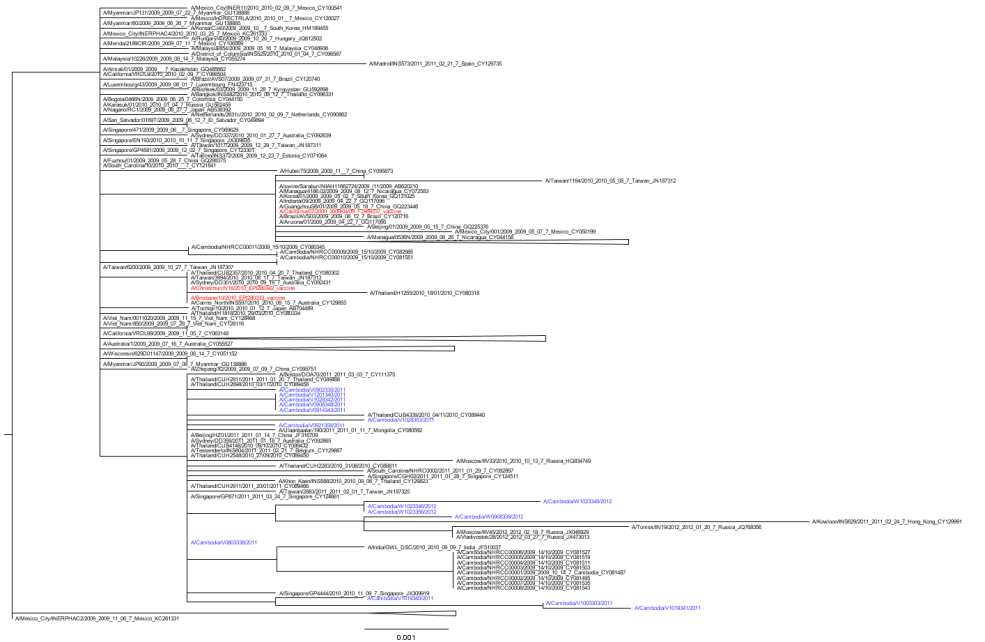

Supplement: S2 Fig — Vaccine strains are highlighted in red. Node support was calculated with aLRT and was >0.70 for all major nodes. All samples fell within the same major clade that included other sequences isolated from Cambodia. (TIF) [file pone.0152529.s002.tif]

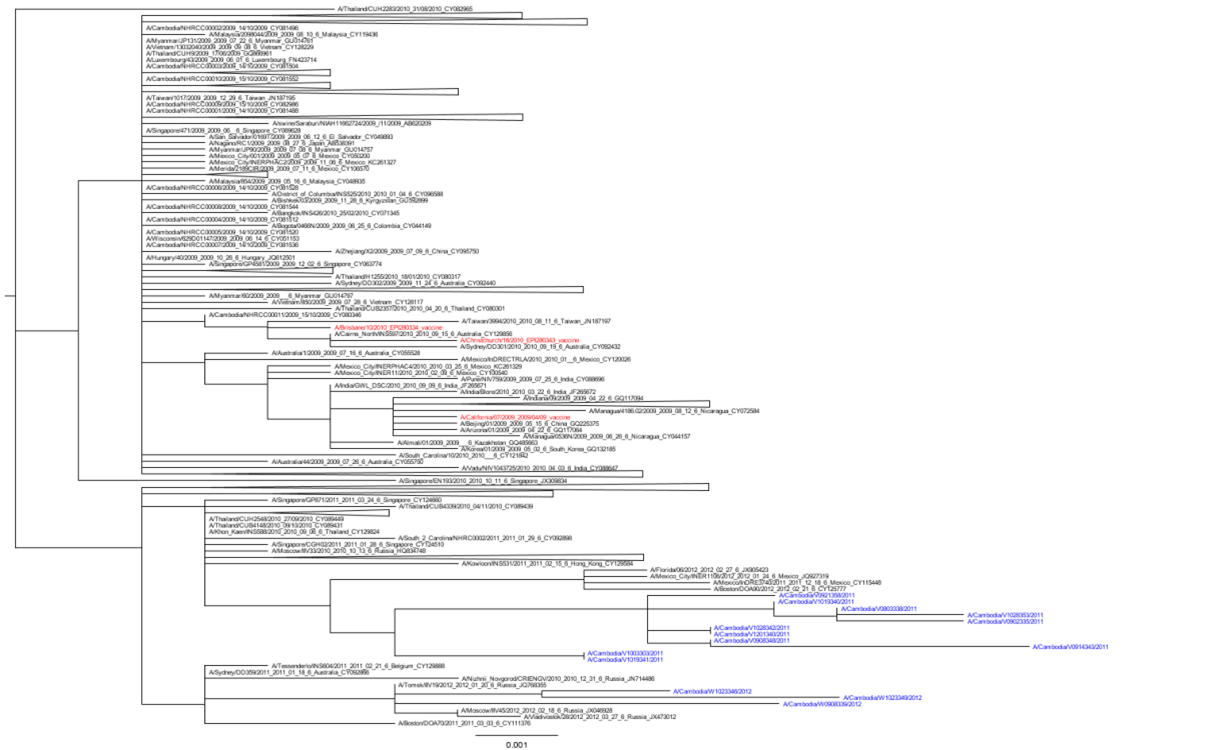

Supplement: S3 Fig — Vaccine strains are highlighted in red. Node support was calculated with aLRT and was >0.70 for all major nodes. The 2011 samples fell within the same clade and 2012 samples fell within a different clade. (TIF) [file pone.0152529.s003.tif]

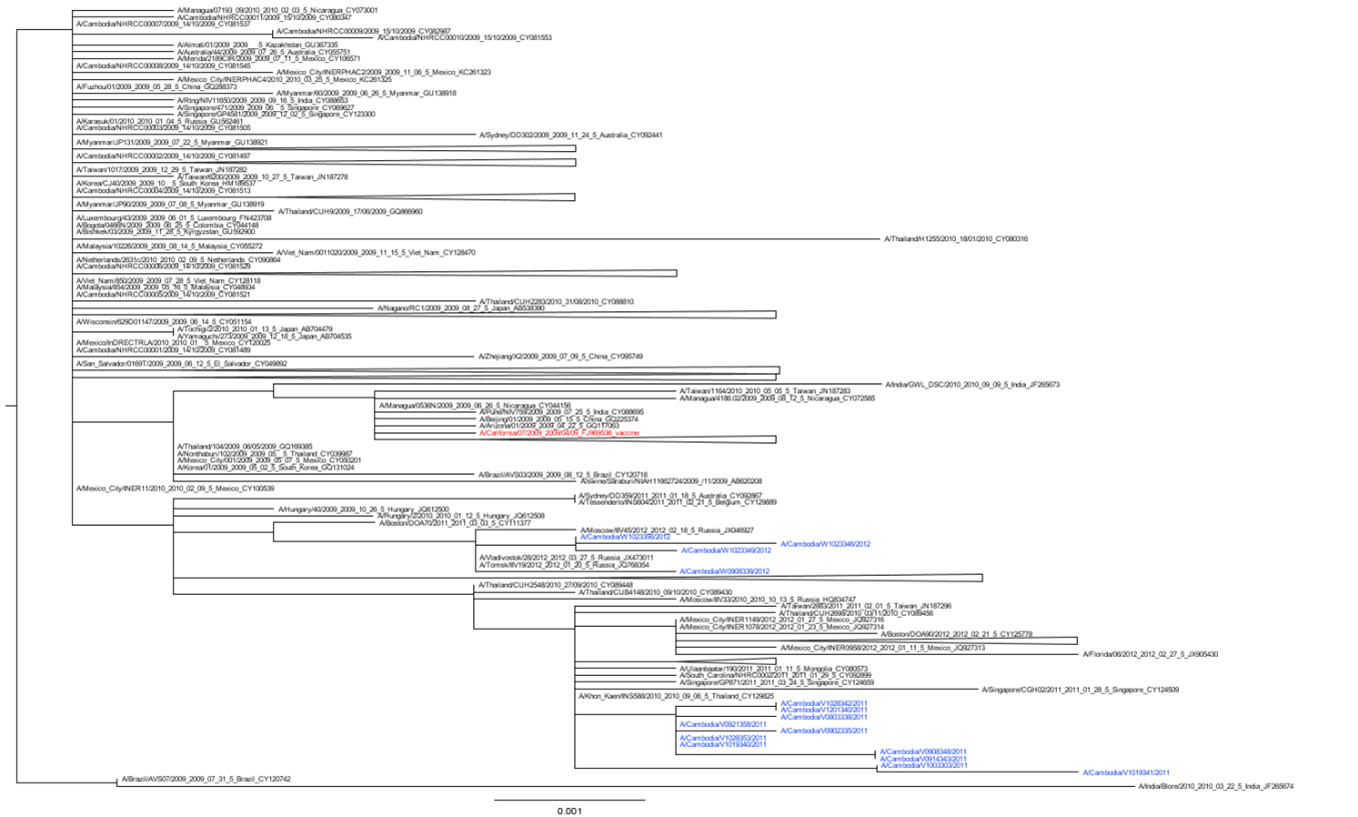

Supplement: S4 Fig — Vaccine strains are highlighted in red. Node support was calculated with aLRT and was >0.70 for all major nodes. The 2011 samples fell within the same clade and 2012 samples fell within a different clade. (TIF) [file pone.0152529.s004.tif]

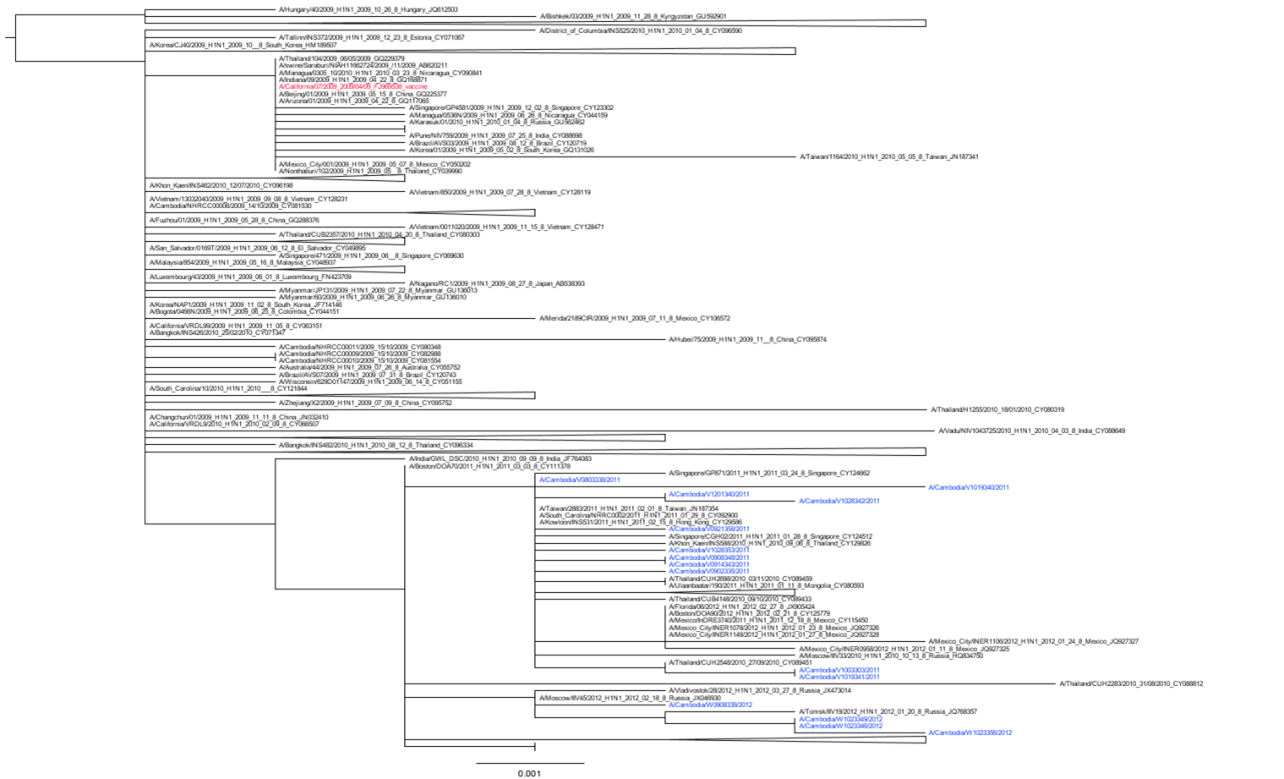

Supplement: S5 Fig — Vaccine strains are highlighted in red. Node support was calculated with aLRT and was >0.70 for all major nodes. The 2011 samples fell within the same clade and 2012 samples fell within a different clade. (TIF) [file pone.0152529.s005.tif]
