# Supplementary material for: Human Sentinel Surveillance of Influenza and Other Respiratory Viral Pathogens in Border Areas of Western Cambodia
Source: PLoS One. 2016 Mar 30;11(3):e0152529. doi: 10.1371/journal.pone.0152529 (PMC4814059; doi:10.1371/journal.pone.0152529)
Supplement: S1 Table — (DOCX) [file pone.0152529.s006.docx]

**S1 Table.** Forward and reverse primers used in amplification of influenza viruses with 5' and 3' modifications indicated.

| **Primer/Probe** | **Sequence 5'>3'** | **5'modification** | **3'modification** |
| --- | --- | --- | --- |
| InfA Forward | GAC CRA TCC TGT CAC CTC TGA C | None | None |
| InfA Reverse | AGG GCA TTY TGG ACA AAK CGT CTA | None | None |
| InfA Probe^1^ | TGC AGT CCT CGC TCA CTG GGC ACG |  |  |
| SW InfA Forward | GCA CGG TCA GCA CTT ATY CTR AG | None | None |
| SW InfA Reverse | GTG RGC TGG GTT TTC ATT TGG TC | None | None |
| SW InfA Probe^2^ | CYA CTG CAA GCC CA’T’ ACA CAC AAG CAG GCA | FAM | BHQ1 |
| SW H1 Forward | GTG CTA TAA ACA CCA GCC TYC CA | None | None |
| SW H1 Reverse | CGG GAT ATT CCT TAA TCC TGT RGC | None | None |
| SW HI Probe^2^ | CA GAA TAT ACA ‘T’ CC RGT CAC AAT TGG ARA A | FAM | BHQ1 |
| RnaseP Forward | AGA TTT GGA CCT GCG AGC G | None | None |
| RnaseP Reverse | GAG CGG CTG TCT CCA CAA GT | None | None |
| RnaseP Probe^1^ | TTC TGA CCT GAA GGC TCT GCG CG | None | None |
| pdm InfA For Primer | TTG CAG TAG CAA GTG GGC ATG A | None | None |
| pdm InfA Rev Primer | TCT TGT GAG CTG GGT TTT CAT TTG | None | None |
| pdm InfA Probe ^2^ | TGA ATG GGT C”T”A TCC CGA CCA GTG AGT AC | FAM | BHQ1 |
| pdm H1 For Primer^3^ | GTG CTA TAA ACA CCA GCC TCC CAT T | None | None |
| pdm H1 Rev Primer^3^ | AGA CGG GAY ATT CCT CAA TCC TG | None | None |
| pdm H1 Probe ^2,3^ | ATA CAT CCG A”T”C ACM ATT GGA AAA TGT CC | FAM | BHQ1 |
| pdm H1 For Primer (GRswH1-349Fw) | GAG CTA AGA GAG CAA TTG A | None | None |
| pdm H1 Rev Primer  (GRswH1-601Rv) | GTA GAT GGA TGG TGA ATG | None | None |
| pdm H1 Probe  GRswH1-538Probe(-)) | TTC CTG AGC TTT GGG TAT GA | None | None |
| FluA Forward^4^ | GAC CRA TCC TGT CAC CTC TGA C | None | None |
| FluA Rerverse^4^ | AGG GCA TTY TGG ACA AAK CGT CTA | None | None |
| FluA Probe^2, 4^ | TGC AGT CCT CGC TCA CTG GGC ACG | FAM | BHQ1 |
| FluB Forward^5^ | TCC TCA AYT CAC TCT TCG AGC G | None | None |
| FluB Rerverse^5^ | CGG TGC TCT TGA CCA AAT TGG | None | None |
| FluB Probe^2, 5^ | CCA ATT CGA GCA GCT GAA ACT GCG GTG | FAM | BHQ1 |
| A H1 Forward^3^ | AAC TAC TAC TGG ACT  CTR CTK GAA | None | None |
| A H1 Rerverse^3^ | CCA TTG GTG CAT TTG AGK TGA TG | None | None |
| A H1 Probe^2,3^ | TGA YCC AAA GCC TCT ACT CAG TGC GAA AGC | FAM | BHQ1 |
| A H3 Forward^3^ | AAG CAT TCC YAA TGA CAA ACC | None | None |
| A H3 Rerverse^3^ | ATT GCR CCR AAT ATG CCT CTA GT | None | None |
| A H3 Probe^2, 3^ | CAG GAT CAC ATA TGG GSC CTG TCC CAG | FAM | BHQ1 |
| A H5a Forward^3^ | TGG AAA GTR TAA RAA ACG GAA CGT | None | None |
| A H5a Rerverse^3^ | YGC TAG GGA RCT CGC CAC TG | None | None |
| A H5a Probe1^2, 3^ | TGA CTA CCC GCA GTA TTC AGA AGA AGC AAG ACT AA | FAM | BHQ1 |
| A H5a Probe2^2^ | CAA CTA TCC GCA GTA TTC AGA AGA AGC AAG ATT AA | FAM | BHQ1 |
| pdm InfA Forward | TTG CAG TAG CAA GTG GGC ATG A | None | None |
| pdm InfA Rerverse | TCT TGT GAG CTG GGT TTT CAT TTG | None | None |
| pdm InfA Probe^2^ | TGA ATG GGT CTA TCC CGA CCA GTG AGT AC | FAM | BHQ1 |

^1^ TaqMan® probes are labeled at the 5'end with the reporter molecule 6-carboxyfuorescein (FAM) and with the quencher, Blackhole Quencher 1 (BHQ1) (Biosearch Technologies, Inc., Novato, CA) at the 3'end.

^2^ TaqMan® probes are labeled at the 5'end with the reporter molecule 6-carboxyfuorescein (FAM) and quenched internally with a modified ‘T’residue with BHQ1, with a modified 3'end to prevent probe extension by Taq polymerase.

^3^ HA gene

^4^ M gene

^5^ NS gene
