# Supplementary material for: Human Sentinel Surveillance of Influenza and Other Respiratory Viral Pathogens in Border Areas of Western Cambodia
Source: PLoS One. 2016 Mar 30;11(3):e0152529. doi: 10.1371/journal.pone.0152529 (PMC4814059; doi:10.1371/journal.pone.0152529)
Supplement: S2 Table — (DOCX) [file pone.0152529.s007.docx]

**S2 Table.** Samples collected for genetic analysis in this study including virus subtype, sample origin, specimen type, sampling date and segments successfully sequenced.

| **Sample** | **Virus Subtype^a^** | **Sample Origin** | **Specimen** | **Sampling Date** | **Segments Sequenced^b,c^** |
| --- | --- | --- | --- | --- | --- |
| V0921358 | pH1N1 | Battambang | Combined Nasal and Throat | 9/16/2011 | PB2, PB1, PA, HA, NP, NA, MP, NS |
| V1019341 | pH1N1 | Oddar Meanchey | Combined Nasal and Throat | 10/11/2011 | PB2, PB1, PA, HA, NP, NA, MP, NS |
| V1028342 | pH1N1 | Battambang | Combined Nasal and Throat | 10/18/2011 | PB2, PB1, PA, HA*, NP, NA*, MP, NS |
| V1201340 | pH1N1 | Battambang | Combined Nasal and Throat | 11/21/2011 | PB2, PB1, PA, HA*, NP, NA*, MP, NS |
| V0803338 | pH1N1 | Oddar Meanchey | Combined Nasal and Throat | 7/25/2011 | PB2, PB1, PA, HA, NP, NA*, MP, NS |
| V0902335 | pH1N1 | Oddar Meanchey | Combined Nasal and Throat | 8/25/2011 | PB2, PB1, PA, HA, NP, NA, MP, NS |
| V0908348 | pH1N1 | Oddar Meanchey | Combined Nasal and Throat | 8/31/2011 | PB2, PB1, PA, HA, NP, NA*, MP, NS |
| V0914343 | pH1N1 | Oddar Meanchey | Combined Nasal and Throat | 9/6/2011 | PB2, PB1, PA, HA*, NP, NA*, MP, NS |
| V1003303 | pH1N1 | Oddar Meanchey | Combined Nasal and Throat | 9/22/2011 | PB2, PB1, PA, HA, NP, NA*, MP, NS |
| V1019340 | pH1N1 | Oddar Meanchey | Combined Nasal and Throat | 10/10/2011 | PB2, PB1, PA, HA, NP, NA*, MP, NS |
| V1028353 | pH1N1 | Pailin | Combined Nasal and Throat | 10/21/2011 | PB2, PB1, PA, HA, NP, NA*, MP, NS |
| V1012345 | H3N2 | Battambang | Combined Nasal and Throat | 10/4/2011 | PB2, PB1, PA, HA, NP, NA, MP, NS |
| V1028340 | H3N2 | Battambang | Combined Nasal and Throat | 10/18/2011 | PB2, PB1, PA, HA, NP, NA, MP, NS |
| V1221301 | H3N2 | Oddar Meanchey | Combined Nasal and Throat | 10/14/2011 | PB2, PB1, PA, HA, NP, NA, MP, NS |
| V1028352 | H3N2 | Pailin | Combined Nasal and Throat | 10/18/2011 | PB2, PB1, PA, HA, NP, NA, MP, NS |
| W0908339 | pH1N1 | Pailin | Combined Nasal and Throat | 7/18/2012 | HA*, NP, NA*, MP, NS |
| W1023346 | pH1N1 | Battambang | Combined Nasal and Throat | 10/8/2012 | HA, NP, NA*, MP, NS |
| W1023349 | pH1N1 | Banteay Meanchey | Combined Nasal and Throat | 10/11/2012 | HA*, NP, NA*, MP, NS |
| W1023356 | pH1N1 | Battambang | Combined Nasal and Throat | 10/19/2012 | NP, MP, NS |
| W0908340 | H3N2 | Battambang | Combined Nasal and Throat | 8/28/2012 | HA, NP, NA, MP, NS |
| W0921311 | H3N2 | Oddar Meanchey | Combined Nasal and Throat | 9/10/2012 | HA, NP, NA, MP, NS |
| W1023343 | H3N2 | Oddar Meanchey | Combined Nasal and Throat | 10/5/2012 | HA, NP, NA, MP, NS |
| W1023347 | H3N2 | Oddar Meanchey | Combined Nasal and Throat | 10/10/2012 | HA, NP, NA, MP, NS |
| W1023353 | H3N2 | Oddar Meanchey | Combined Nasal and Throat | 10/18/2012 | HA, NP, NA, MP, NS |
| W1023355 | H3N2 | Oddar Meanchey | Combined Nasal and Throat | 10/18/2012 | HA, NP, NA, MP, NS |

^a^ All viral types Influenza A.

^b^ Segments marked with an asterisk are partial sequences.

^c^ Segments where sequence data was not obtained are not listed.
