# Supplementary material for: Human Sentinel Surveillance of Influenza and Other Respiratory Viral Pathogens in Border Areas of Western Cambodia
Source: PLoS One. 2016 Mar 30;11(3):e0152529. doi: 10.1371/journal.pone.0152529 (PMC4814059; doi:10.1371/journal.pone.0152529)
Supplement: S3 Table — The modifications were made by following the alignments of VP1 sequences of different EV71 strains collected during 2002–2011 from Thailand, Taiwan, Philippines, Vietnam, and China available in Genbank including the sequences with the accession No. JN191177-9, FJ969151, FJ969163, JQ 621835, JQ621841, AM490141-63, JQ315092, and JX203305. The primers for simultaneous detection of enteroviruses and rhinoviruses were designed in the polyprotein gene, between 5′ non-coding region (5′NCR) and VP4/VP2 regions that was previously described by Coiras et al. 2004 [29]. EV/RV-2n was modified from primer 2-EV/RV [29] for using in nested PCR reaction. (DOCX) [file pone.0152529.s008.docx]

| **S3 Table**. Primers for RT-PCR and nucleotide sequencing for EV/RV and EV71: The enterovirus 71 type specific primers were modified from the previous study by Singh et al. (2002) [[50](#_ENREF_50)]. The modifications were made by following the alignments of VP1 sequences of different EV71 strains collected during 2002-2011 from Thailand, Taiwan, Philippines, Vietnam, and China available in GenBank including the sequences with the accession No. JN191177-9, FJ969151, FJ969163, JQ 621835, JQ621841, AM490141-63, JQ315092, and JX203305. The primers for simultaneous detection of enteroviruses and rhinoviruses were designed in the polyprotein gene, between 5′ non-coding region (5′NCR) and VP4/VP2 regions that was previously described by Coiras et al. 2004 [[26](#_ENREF_26)]. EV/RV-2n was modified from primer 2-EV/RV [[26](#_ENREF_26)] for using in nested PCR reaction. | | | | | |
| --- | --- | --- | --- | --- | --- |
| **Primer** | **Sequence (5'-3')** | **Gene** | **Gene position** | | **PCR product size (bp)** |
| EV71-VP1f | GTYCTYAACTCRCACAGYA | VP1 | | 205-223^a^ |  |
| EV71-VP1r | TTRACAAAAACTGARGGGTT |  | | 526-545^a^ | 341 |
| EV71-VP1r2 | CCRGTRGGHGTRCACGCAAC |  | | 412-431^a^ | 227 |
| 1-EV/RV | CTCCGGCCCCTGAATRYGGCTAA | 5'NCR-VP4/VP2 | | 447-469^a^ 436-458^b^ |  |
| 2-EV/RV | TCIGGIARYTTCCASYACCAICC |  | | 1178-1200^a^ 1046-1068^b^ | 754 (EV) 633 (RV) |
| 3-EV/RV | ACCRASTACTTTGGGTRWCCGTG |  | | 538-560^a^ 527-549^b^ |  |
| EV/RV-2n | CCGGYAAYTTCCACCACCA |  | | 1181-1199^a^ 1049-1067^b^ | 662 (EV) 541 (RV) |
| ^a^Gene position is based on the sequence of Human enterovirus 71 strain H, GenBank accession No. AY053402 | | | | | |
| ^b^Gene position is based on the sequence of Human rhinovirus A2, GenBank accession No. X02316 | | | | | |
