# Supplementary material for: Human Sentinel Surveillance of Influenza and Other Respiratory Viral Pathogens in Border Areas of Western Cambodia
Source: PLoS One. 2016 Mar 30;11(3):e0152529. doi: 10.1371/journal.pone.0152529 (PMC4814059; doi:10.1371/journal.pone.0152529)
Supplement: S4 Table — (DOCX) [file pone.0152529.s009.docx]

| **S4 Table**. Total number of viruses identified by pan-enteroviruses and rhinoviruses (EV/RV) PCR and nucleotide sequencing^†^ | |
| --- | --- |
|  |  |
| **Results** | **Number of cases** |
| Human coxsackievirus-A | 10 |
| Human coxsackievirus-B | 2 |
| Human echovirus | 5 |
| Human rhinovirus-A | 11 |
| Human rhinovirus-B | 4 |
| Human rhinovirus-C | 3 |
| Human rhinovirus-unknown species^‡^ | 15 |
| Negative | 114 |
| **Total** | **164** |
| ^†^Good quality sequences with the various size between 433-625 bp were subjected for homology searches using nucleotide BLAST program | |
| ^‡^The samples were weak positive for human rhinovirus by EV/RV PCR, but undetectable by sequencing. | |
