# Supplementary material for: Human Sentinel Surveillance of Influenza and Other Respiratory Viral Pathogens in Border Areas of Western Cambodia
Source: PLoS One. 2016 Mar 30;11(3):e0152529. doi: 10.1371/journal.pone.0152529 (PMC4814059; doi:10.1371/journal.pone.0152529)
Supplement: S5 Table — (DOCX) [file pone.0152529.s010.docx]

**S5 Table**. pH1N1 average percent nucleotide sequence identity between vaccine strains and Cambodia isolates gene segments.

| **Gene** | **%ntid^a^ (A/California/7/2009)** | **%ntid^a^ (A/Brisbane/10/2010)** | **%ntid^a^ (A/Christchurch/16/2010)** |
| --- | --- | --- | --- |
| HA | 98.49 | 98.67 | 98.34 |
| NP | 99.23 | ^b^ | ^b^ |
| NA | 98.61 | 98.61 | 98.45 |
| MP | 99.50 | 99.60 | 99.60 |
| NS | 99.35 | ^b^ | ^b^ |

^a^ Percent nucleotide identity

^b^ Segments unavailable for vaccine strain.
