# Supplementary material for: Human Sentinel Surveillance of Influenza and Other Respiratory Viral Pathogens in Border Areas of Western Cambodia
Source: PLoS One. 2016 Mar 30;11(3):e0152529. doi: 10.1371/journal.pone.0152529 (PMC4814059; doi:10.1371/journal.pone.0152529)
Supplement: S6 Table — AA substitution nomenclature is as follows; reference amino acid (A/California/7/2009), amino acid site, sample amino acid. Amino acids are numbered from the start codon of the segment (ATG:Methionine). (DOCX) [file pone.0152529.s011.docx]

**S6 Table.** Changes in glycosylation, antigenic and polymorphic sites linked to AA substitutions and sub-antigenic/catalytic sites (epitopes) where amino acid (AA) substitutions were found for for the HA and NA segments of pH1N1. AA substitution nomenclature is as follows; reference amino acid (A/California/7/2009), amino acid site, sample amino acid. Amino acids are numbered from the start codon of the segment (ATG:Methionine).

| **Segment** | **AA Substitution^a^** | **Notation** |
| --- | --- | --- |
| HA^b^ | E391K | IFA Marker |
|  | N277D/G | Polymorphic site |
|  | H155R | Ca Antigenic site ^d^ |
|  | G172E | Sb antigenic site^d^ |
|  | S200P | Improved growth in MDCK cell culture and eggs^e^ |
|  | N173D | Sa antigenic site ^d^ |
| NA^c^ | N248D | Antigenic site^f^ |
|  | N44S | Addition of glycosylation site (NQN to NQS) |
|  | D451G | Polymorphic site |
|  | N63S | Loss of glycosylation site (NQT to SQT) |
|  | I396V | Antigenic site^f^ |

AA: amino acid

^a^ Amino acid of reference on left, sample substitution on right of amino acid position number.

^b^ HA numbering starts from Methionine as position 1. Partial sequences were analyzed, alignment therefore starts from amino acid 8-553 (first 7 aa missing).

^c^NA numbering starts from Methionine as position 1. Partial sequences were analyzed, alignment therefore starts from amino acid 33-433 (first 32 aa missing).

^d^ Chutinimitkul S, Chieochansin T, Payungporn S, Samransamruajkit R, Hiranras T, et al. (2008) Molecular characterization and phylogenetic analysis of H1N1 and H3N2 human influenza A viruses among infants and children in Thailand. Virus Res 132: 122-131.

^e^ Suphaphiphat P, Franti M, Hekele A, Lilja A, Spencer T, et al. (2010) Mutations at positions 186 and 194 in the HA gene of the 2009 H1N1 pandemic influenza virus improve replication in cell culture and eggs. Virol J 7: 157.

^f^ Wan H, Gao J, Xu K, Chen H, Couzens LK, et al. (2013) Molecular basis for broad neuraminidase immunity: conserved epitopes in seasonal and pandemic H1N1 as well as H5N1 influenza viruses. J Virol 87: 9290-9300.
