# Supplementary material for: Human Sentinel Surveillance of Influenza and Other Respiratory Viral Pathogens in Border Areas of Western Cambodia
Source: PLoS One. 2016 Mar 30;11(3):e0152529. doi: 10.1371/journal.pone.0152529 (PMC4814059; doi:10.1371/journal.pone.0152529)
Supplement: S7 Table — AA substitution nomenclature is as follows; reference amino acid (A/California/7/2009), amino acid site, sample amino acid. Amino acids are numbered from the start codon of the segment (ATG:Methionine). (DOCX) [file pone.0152529.s012.docx]

**S7 Table**. Unique pH1N1 amino acid (AA) changes of unknown function in specific samples for the NA gene as compared to A/California/7/2009. AA substitution nomenclature is as follows; reference amino acid (A/California/7/2009), amino acid site, sample amino acid. Amino acids are numbered from the start codon of the segment (ATG:Methionine).

| **Sample** | **AA Substitution^a,b^** |
| --- | --- |
| V1003303 | I34V |
| V1019341 | I34V |
| V0914343 | Q43R |
|  | N59Y |
|  | I108F |
|  | D451N |
| W0908339 | I216F |
| W1023349 | V264I |
|  | N270S |
| V1028353 | F445L |
| 0908348 | D451N |

AA: amino acid

^a^ Amino acid of reference (A/California/7/2009) on left, sample substitution on right of amino acid position number.

^b^ NA numbering starts from Methionine as position 1. Partial sequences were analyzed, alignment therefore starts from amino acid 33-433 (first 32 aa missing).
