# Supplementary material for: Human Sentinel Surveillance of Influenza and Other Respiratory Viral Pathogens in Border Areas of Western Cambodia
Source: PLoS One. 2016 Mar 30;11(3):e0152529. doi: 10.1371/journal.pone.0152529 (PMC4814059; doi:10.1371/journal.pone.0152529)
Supplement: S8 Table — (DOCX) [file pone.0152529.s013.docx]

**S8 Table.** pH1N1 sample amino acid substitution summary and selection analysis by group per segment analyzed.

| **Gene** | **Sample** | **No. of samples** | **AA changes relative to A/California/7/2009** | **AA subst.**  **w/in grp** | **dS** | **dN** | **dN/dS** | **Selection** |
| --- | --- | --- | --- | --- | --- | --- | --- | --- |
| **HA^b^** | 2011^a^ | 11 | 20 | 12 | .013 | .002 | .154 | Purifying |
|  | 2012 | 3 | 14 | 6 | .016 | .003 | .188 | Purifying |
|  | All | 14 | 26 | 21 | .031 | .004 | .129 | Purifying |
| **NA^c^** | 2011^a^ | 11 | 19 | 13 | .008 | .004 | .500 | Purifying |
|  | 2012 | 3 | 13 | 6 | .016 | .004 | .250 | Purifying |
|  | All | 14 | 25 | 21 | .015 | .006 | .400 | Purifying |
| **MP** | 2011^a^ | 11 | 4 | 3 | .009 | .001 | .111 | Purifying |
|  | 2012 | 4 | 4 | 3 | .004 | .002 | .500 | Purifying |
|  | All | 15 | 7 | 6 | .010 | .002 | .200 | Purifying |
| **NP** | 2011^a^ | 11 | 3 | 1 | .007 | .0002 | .029 | Purifying |
|  | 2012 | 4 | 4 | 2 | .005 | .001 | .200 | Purifying |
|  | All | 15 | 5 | 3 | .015 | .0004 | .027 | Purifying |
| **NS** | 2011^a^ | 11 | 9 | 8 | .003 | .003 | 1.00 | Neutral |
|  | 2012 | 4 | 3 | 2 | .004 | .002 | .500 | Purifying |
|  | All | 15 | 11 | 10 | .007 | .004 | .571 | Purifying |

**^a^** All samples found within the Cambodia 2011 clade (See Figure 1).

**^b^** Partial only, covers nucleotides 22-1683 (553 amino acids).

**^c^** Partial only, covers nucleotides 97-1396 (433 amino acids).

**AA:** amino acid

**NT**: nucleotide

**dS**: number of synonymous substitutions per site taking into account the proportion of synonymous differences.

**dN**: number of nonsynonymous substitutions per site taking into account the proportion of nonsynonymous differences.

**dN/dS**: Relative measure of selection. dN/dS < 1, purifying selection, dN/dS > 1, positive selection, dN/dS ~ 1, neutral selection.
