# Supplementary material for: Human Sentinel Surveillance of Influenza and Other Respiratory Viral Pathogens in Border Areas of Western Cambodia
Source: PLoS One. 2016 Mar 30;11(3):e0152529. doi: 10.1371/journal.pone.0152529 (PMC4814059; doi:10.1371/journal.pone.0152529)
Supplement: S9 Table — (DOCX) [file pone.0152529.s014.docx]

**S9 Table.** H3N2 average percent nucleotide sequence identity between vaccine strains and Cambodia isolates gene segments.

| **Gene** | **%ntid^a^**  **(A/Perth/16/2009)** | **%ntid^a^ (A/Perth/10/2010)** | **%ntid^a^ (A/Victoria/361/2011)** | **%ntid^a^**  **(A/Ohio/02/2012)** |
| --- | --- | --- | --- | --- |
| HA | 98.40 | 98.23 | 99.10 | 98.93 |
| NA | 98.36 | 99.06 | 99.13 | 99.39 |
| MP | 99.22 | 99.22 | 99.66 | 99.40 |
| NS | 98.70 | 98.40 | 99.29 | ^b^ |

^a^ Percent nucleotide identity

^b^ Segments unavailable for vaccine strain.
