# Supplementary material for: Human Sentinel Surveillance of Influenza and Other Respiratory Viral Pathogens in Border Areas of Western Cambodia
Source: PLoS One. 2016 Mar 30;11(3):e0152529. doi: 10.1371/journal.pone.0152529 (PMC4814059; doi:10.1371/journal.pone.0152529)
Supplement: S10 Table — (DOCX) [file pone.0152529.s015.docx]

**S10 Table.** H3N2 sample amino acid substitution summary and selection analysis by group per segment analyzed.

| **Gene** | **Sample** | **No. of samples** | **AA changes relative to A/Victoria/361/2011** | **AA subst.**  **w/in grp** | **dS** | **dN** | **dN/dS** | **Selection** |
| --- | --- | --- | --- | --- | --- | --- | --- | --- |
| **HA** | 2011 | 4 | 11 | 7 | .009 | .003 | .333 | Purifying |
|  | 2012 | 6 | 20 | 15 | .013 | .005 | .385 | Purifying |
|  | All | 10 | 23 | 19 | .016 | .006 | .375 | Purifying |
| **MP** | 2011 | 4 | 1 | 1 | .008 | .001 | .125 | Purifying |
|  | 2012 | 6 | 2 | 2 | .011 | .001 | .091 | Purifying |
|  | All | 10 | 3 | 3 | .013 | .001 | .077 | Purifying |
| **NA** | 2011 | 4 | 6 | 4 | .013 | .002 | .154 | Purifying |
|  | 2012 | 6 | 12 | 10 | .009 | .003 | .333 | Purifying |
|  | All | 10 | 15 | 13 | .015 | .003 | .200 | Purifying |
| **NS** | 2011 | 4 | 7 | 6 | .004 | .005 | 1.25 | Neutral |
|  | 2012 | 6 | 7 | 6 | .004 | .006 | 1.50 | Neutral |
|  | All | 10 | 12 | 11 | .009 | .005 | .556 | Purifying |

**AA:** amino acid

**NT**: nucleotide

**dS**: number of synonymous substitutions per site taking into account the proportion of synonymous differences.

**dN**: number of nonsynonymous substitutions per site taking into account the proportion of nonsynonymous differences.

**dN/dS**: Relative measure of selection. dN/dS < 1, purifying selection, dN/dS > 1, positive selection, dN/dS ~ 1, neutral selection.
