# Supplementary material for: Human Sentinel Surveillance of Influenza and Other Respiratory Viral Pathogens in Border Areas of Western Cambodia
Source: PLoS One. 2016 Mar 30;11(3):e0152529. doi: 10.1371/journal.pone.0152529 (PMC4814059; doi:10.1371/journal.pone.0152529)
Supplement: S11 Table — AA substitution nomenclature is as follows; reference amino acid (A/Victoria/361/2011), amino acid site, sample amino acid. Amino acids are numbered from the start codon of the segment (ATG:Methionine). (DOCX) [file pone.0152529.s016.docx]

**S11 Table**. Unique H3N2 amino acid changes of unknown function to specific to samples for the HA gene as compared to A/Victoria/361/2011. AA substitution nomenclature is as follows; reference amino acid (A/Victoria/361/2011), amino acid site, sample amino acid. Amino acids are numbered from the start codon of the segment (ATG:Methionine).

| **Sample** | **AA Substitution^a,b^** |
| --- | --- |
| W1023343 | A16S |
| W1023347 | A16S |
| W0921311 | P237L |
| W0908340 | A288S |
|  | Y527H |

AA: amino acid

^a^ Amino acid of reference (A/Victoria/361/2011) on left, sample substitution on right of amino acid position number.

^b^ HA numbering starts from Methionine as position 1.
