# Supplementary material for: Human Sentinel Surveillance of Influenza and Other Respiratory Viral Pathogens in Border Areas of Western Cambodia
Source: PLoS One. 2016 Mar 30;11(3):e0152529. doi: 10.1371/journal.pone.0152529 (PMC4814059; doi:10.1371/journal.pone.0152529)
Supplement: S12 Table — AA substitution nomenclature is as follows; reference amino acid (A/Victoria/361/2011), amino acid site, sample amino acid. Amino acids are numbered from the start codon of the segment (ATG:Methionine). (DOCX) [file pone.0152529.s017.docx]

**S12 Table**. Unique H3N2 amino acid changes of unknown function to specific to samples for the NA gene as compared to A/Victoria/361/2011. AA substitution nomenclature is as follows; reference amino acid (A/Victoria/361/2011), amino acid site, sample amino acid. Amino acids are numbered from the start codon of the segment (ATG:Methionine).

| **Sample** | **AA Substitution^a,b^** |
| --- | --- |
| W0921311 | M15V |
|  | P55Q |
| W1023355 | T72I |
|  | A462T |
| W1023353 | I77M |
| W1023347 | T267K |
| V1012345 | I443V |

AA: amino acid

^a^ Amino acid of reference (A/Victoria/361/2011) on left, sample substitution on right of amino acid position number.

^b^ NA numbering starts from Methionine as position 1.
