# Supplementary material for: Human Sentinel Surveillance of Influenza and Other Respiratory Viral Pathogens in Border Areas of Western Cambodia
Source: PLoS One. 2016 Mar 30;11(3):e0152529. doi: 10.1371/journal.pone.0152529 (PMC4814059; doi:10.1371/journal.pone.0152529)
Supplement: S13 Table — Sequencing results of the EV71 isolates based on (A) pan rhinovirus/enterovirus inner primer and (B) Sequencing results of the EV71 isolates based on an enterovirus 71 inner primer. (DOCX) [file pone.0152529.s018.docx]

**S13 Table.** Sequencing results of the EV71 isolates based on (A) pan rhinovirus/enterovirus inner primer and (B) Sequencing results of the EV71 isolates based on an enterovirus 71 inner primer

1. /TATTCACTGAAACATCCGGGCGCGTTGGTTTATCCACTGCTGTAGCGTCAGAATCTGAGCAGTAGGAAGGCCACTCACCATAACCGACTATAATGTTAGCCGCTTCTTGCGTGGTGATGGTGGAGTTGCCAATAGTTAATTGTGCCACTCGATCACTGTATCCACATGCCTCAGCGGATGGAGACTTCAGTGGCGCTGCCATTTCAGTGAAGATGTCTTTAACAGGATTTGCAAACTTGTCTGGATCCTGCTTGAGACTCTGTTTGCCTGCTGTGGCAGCATAAGAGTCTTTGTAGTAATTGATGGTGGTGTAGTTTATGGTAGAACCCTCAGTGGCTGAGTTTGAGTTCTCGTGTGAACCGGAGCGCTGTGTAGACACTTGCGAACCCATGTTTAGTTGCGTTAAGGGTCAAAATATAATCAAGGGTGGTCACAGAATTCAAGGTTAATGGTACAAAACCAATAAATTGGTAAATAATTGCTCTGTTGCACACCGGATGGCCAATCCAATAGCTATATGGTAACAAT
2. /CGGGATTAGTTGGAGAGATAGATCTCCCTCTTGAGGGCACAACTAACCCAAATGGTTACGCCAATTGGGACATAGATATAACAGGTTACGCGCAAATGCGTAGAAAGGTAGAACTATTCACCTACATGCGC
